# Research on the implementation path of digital-intelligent healthcare based on the TAM model from the perspective of high-quality development

**DOI:** 10.1186/s12913-026-14433-1

**Published:** 2026-03-27

**Authors:** Zijue Qi, Huize Han, Yidayetula Abuduaini, Aikeda Litipu, Jun Li

**Affiliations:** School of Public Health, Capital Medical University, Beijing, 100069 China

**Keywords:** Technology acceptance model, TAM, Digital-intelligent healthcare, High-quality development, Structural equation modeling, Mixed-methods

## Abstract

**Background:**

Digital-intelligent healthcare is essential for high-quality, accessible medical services, yet its adoption is hindered by varying public attitudes.

**Objective:**

This study investigates determinants of public acceptance and explores reasons for suboptimal implementation to inform sustainable, patient-centered pathways.

**Methods:**

We employed a sequential mixed-methods design grounded in the Technology Acceptance Model. A survey collected 456 valid responses, with 340 valid for structural equation modeling after data screening, followed by semi-structured interviews with eight purposively selected individuals (clinicians, students, educators, elderly patients) to triangulate findings.

**Results:**

Perceived usefulness (PU) and perceived ease of use (PEOU) significantly influenced behavioral intention (BI). Attitude played a complete mediating role between PU and BI (indirect effect = 0.784, *p* < 0.01). PU was strongly predicted by subjective norms (β = 0.836, *p* < 0.01) and social influence (β = 0.254, *p* < 0.01). Notably, PEOU exhibited a significant negative direct effect on BI (β = -0.245, *p* < 0.01), suggesting a “trust threshold” where excessive simplification may undermine confidence in high-stakes medical contexts. Qualitative data corroborated these findings, revealing concerns over data security, operational complexity, and inadequate age-friendly design.

**Conclusion:**

Public acceptance of digital-intelligent healthcare is a socio-technical process mediated by attitude and constrained by trust barriers. Effective implementation requires multi-level strategies: alleviating individual technology anxiety, fostering organizational digital leadership, and building a collaborative policy-technology-society network to achieve truly patient-centered, high-quality development.

**Supplementary Information:**

The online version contains supplementary material available at 10.1186/s12913-026-14433-1.

## Introduction

### Basic concepts

High-quality development is a comprehensive concept encompassing economic, social, environmental, and other domains. It aims to achieve economic growth while simultaneously ensuring social equity, enhancing environmental quality, and promoting comprehensive sustainable development. In the field of digital-intelligent healthcare, “high-quality development” signifies enhancing the quality, efficiency, and accessibility of medical services through the introduction and application of digital and intelligent technologies, thereby achieving comprehensive, balanced, and sustainable development of healthcare services.

There are three levels of digital-intelligent healthcare: digitalization, intelligence, and smartification. Digitalization involves transforming real-world information into digital formats for processing. Intelligentization builds upon digitalization by utilizing technologies such as artificial intelligence (AI) and machine learning to enable autonomous decision-making and system optimization. Smartification represents a higher level of integration of data and intelligent technologies to construct complex systems and platforms. As an important development direction in the healthcare sector in the new era, digital-intelligent healthcare integrates advanced technologies such as big data and artificial intelligence. It aims to optimize the allocation of medical resources, improve the quality of healthcare services, and better meet people’s growing needs for healthcare. In recent years, the accelerated integration of new-generation information technologies such as cloud computing, big data, and 5G with medical services has promoted the widespread application of digital-intelligent healthcare in hospitals, increasing service levels while reducing operational costs.

The technology acceptance model (TAM) is a model used to explain individuals’ acceptance and application behaviors toward new technologies and their influencing factors. Its core idea is that an individual’s adoption behavior of new technology is based on their perception and attitude toward the technology. In the TAM, perceived usefulness and perceived ease of use are widely recognized as the main factors influencing an individual’s intention to adopt new technologies. These factors also serve as criteria for evaluating whether a technological service is efficient and accessible, constituting an essential prerequisite for achieving the specific goal of “high-quality development.” For instance, when the public’s perceived ease of use of digital-intelligent healthcare improves, it promotes broader acceptance of the technology, thereby contributing to the high-quality development objective of “service accessibility.” Thus, TAM provides a theoretical foundation for understanding the decision-making process behind individual adoption of new technologies and supports the promotion and application of new technologies within the context of high-quality development goals. In this study, the TAM framework is employed to investigate public acceptance behaviors toward digital-intelligent healthcare applications and their influencing factors, focusing on four dimensions: perceived usefulness, perceived ease of use, subjective norms, and behavioral intention. This approach aims to provide insights into efficient, high-quality, and sustainable pathways for digital-intelligent healthcare and to advance its high-quality development.

### Research background and significance

In the “Key Tasks for Deepening the Medical and Healthcare System Reform in 2024” issued by the State Council of China, the policy related to “Promoting Digital Empowerment of Medical Reform” mentions that carrying out intensive action to achieve interoperability and sharing of information among national medical and health institutions; promoting the development and utilization of public data resources in the health and medical field; and advancing the “handheld handling” and “online handling” of medical service matters. Against the backdrop of rapid development in intelligent technology, the digital and intelligent high-quality development of medical and health service systems has become an important part of thoroughly implementing the “Healthy China” strategy. In this context, the development of digital-intelligent healthcare has achieved phased progress while continuing to undergo exploration. However, during its widespread application, less-than-ideal outcomes persist, among which public attitudes represent a factor that cannot be overlooked.

To explore the potential influencing factors and development challenges of digital-intelligent healthcare, this study employs the Technology Acceptance Model (TAM) to investigate public willingness to adopt digital-intelligent healthcare applications within the context of high‑quality development. The variable design is aligned with the requirements of high‑quality development. A mixed‑method approach combining online and offline questionnaire surveys was adopted, and interviews were conducted from diverse perspectives—including clinicians, medical students, faculty, and elderly individuals—to obtain comprehensive information on the current application status of digital‑intelligent healthcare. By integrating quantitative and qualitative methods, the study offers a relatively holistic scope and demonstrates a degree of innovativeness. Based on the varied attitudes toward digital‑intelligent healthcare among populations with different characteristics, the research provides multi‑faceted and valuable recommendations for its sustainable high‑quality development and related policy formulation. This work further promotes the realization of the “Healthy China” strategy under advancing technology, contributes to more convenient and efficient medical services for the public, and holds progressive significance for the future development of China’s healthcare sector.

### Research status

#### Domestic status

Currently, under the influence of technological advancements in areas such as the internet and AI, digital-intelligent healthcare is beginning to have potential. Its application has transformed the conventional healthcare model, enabling more people to access higher-quality and more convenient medical services. Moreover, this topic has attracted the attention of some scholars. Most existing domestic studies either focus on specific technologies or domains within medical digitalization and intelligentization from the patient perspective [1–3] or merely propose technical and policy application solutions at the hospital level [4–8] (see Table 1). For example, Jiang Qinqin, Zhang Zehong, et al. (2022), in their study “Research on Patients’ Adoption Intention to ‘Internet + Nursing Services’ Based on the Technology Acceptance Model and Theory of Planned Behavior“ [1], which is based on patient satisfaction and the analysis results of TAM and TPB, proposed recommendations for the intelligent development of nursing services. The magazine report “Galloping on the New Track of Smart Healthcare” [4] (2023), against the backdrop of informatization reform, proposed schemes such as the “trinity” model, intelligent surgery platforms, digital twin hospitals, informatization empowering DRGs, and 5G prehospital emergency care to help hospitals advance digital-intelligent healthcare. Currently, relatively few studies have comprehensively explored the application status of digital-intelligent healthcare within the framework of high-quality development. This lack is not conducive to understanding the attitudes and experiences of different population groups towards this technological application and proposing more diverse recommendations, thus exhibiting a certain degree of one-sidedness.

**Table 1:** Current research in China

Author (Year)	Background	Objective(s)	Perspective	Methods	Conclusion
Zhu Change et al.(2019) [9]	The rapid development of hospital intelligent medical system	To study the influence mechanism of the use of hospital intelligent medical system on patients’ satisfaction with medical treatment.	patients	Convenience Sampling, Questionnaire, Technology Acceptance Model, Structural Equation Modelling	Proposing recommendations for the government, hospitals, and system developers to enhance patients’ satisfaction.
Jiang Qinqin et al.(2022) [1]	The “Internet + Nursing Service” was launched.	To analyse the factors influencing patients’ adoption intention to “Internet་Nursing Service”and to provide reference for the future development and decision-making	patients	Convenience Sampling, Questionnaire, Technology Acceptance Model, Theory of Planned Behavior	Proposing recommendations for hospitals, the government, and other stakeholders regarding the ‘Internet + Nursing Service’.”
Jiao Yuelong et al.(2024) [2]	The “Internet + Healthcare” model has become an emerging global trend in the medical industry.	To understand the use of the corresponding scenarios of digital transformation of “convenient medical service” in Shanghai municipal hospital outpatient department from the perspective of patients, and provide relevant suggestions for promoting digital transformation	patients	Questionnaire, Technology Acceptance Model	Proposing recommendations at the hospital and societal levels.
Ma Chengcheng et al.(2024) [3]	The advent of the “big data era” and the advancement of artificial intelligence.	To develop and evaluate an optimized pathway for the integrated prevention and treatment of hypertension and diabetes in primary care settings.	\	\	Proposing recommendations for the development of hospital-based services for hypertension and diabetes.
Guo Xiaoya et al.(2023) [4]	The pace of healthcare informatization development in China is accelerating.	\	\	\	Summarizing and proposing recommendations for hospitals to accelerate the development of informatization and advance the pace of digital-intelligent transformation.
Li Zhaoyang et al.(2023) [5]	The digital transformation of hospitals in China has not yet been achieved.	To explore the methods, steps, and implementation pathways for the digital transformation of hospitals.	\	\	It can be summarized into the following steps: strategy formulation, data governance, technology upgrading, service innovation, organizational change, and management change.
Lv Yingbo et al.(2023) [6]	The development of digital and smart hospitals is underway.	To explore the pathways for leveraging the integration of digital and smart hospitals to drive the high-quality development of public hospitals.	\	\	Facilitating the substantive advancement of digital-intelligent integration in accordance with the State Council’s “Overall Layout Plan for the Construction of Digital China.”
Pan Jie et al.(2024) [7]	The repaid development of digital-intelligent technologies is providing a powerful boost to the high-quality development of the healthcare system.	To analyse the “factor optimization—service optimization—governance optimization” development mechanism driven by digital intelligence and propose corresponding implementation pathways	\	\	Proposing a hospital development path based on a three-tiered framework: factor optimization, service optimization, and governance optimization.
Ruan Hongyi et.al(2025) [8]	The development of smart healthcare	Investigating and applying information management measures to promote the development of modern hospital rehabilitation nursing.	\	\	Proposing optimization strategies focusing on information exchange, resource allocation, and management capacity.

#### International research status

Concurrently, some international scholars have also conducted relevant research; however, the involved fields are relatively narrow. Within the context of population aging, studies have focused primarily on the acceptance of specific digital-intelligent healthcare-related services and devices among the elderly population [10–14]. These include mobile health services, wearable devices, and intelligent monitors, among others. For example, scholars such as Li J et al. [11] (2019) stated in their journal article that, on the basis of research using the TAM and structural equation modelling, perceived usefulness, compatibility, facilitating conditions, and self-reported health status had significant and positive effects on older adults’ intention to use these technologies. In addition, some scholars have approached this topic from the nursing perspective, analysing nurses’ acceptance of the introduction of intelligent technologies. For example, scholars Chen CH and Lee WI [15] (2025) explored the influence of social influence, human–robot trust, and perceived job stress on nurses’ behavioral intention to adopt AI-assisted nursing technologies from a novel perspective and framework.

It can thus be concluded that current research in this field remains relatively one‑dimensional, with a notable gap in studies examining attitudes toward digital‑intelligent healthcare among populations with different characteristics. Under the vision of high‑quality development, public attitudes and adoption intentions are key determinants of the future prospects of digital‑intelligent healthcare. Therefore, this study, based on the Technology Acceptance Model (TAM), collected and analyzed public attitudes toward digital‑intelligent healthcare, and conducted interviews with representatives from diverse demographic groups. By incorporating perspectives from doctors, students, elderly individuals, teachers, and other stakeholders, the research attempts to explore the underlying reasons behind the less‑than‑ideal development of digital‑intelligent healthcare. It further provides an in‑depth examination and discussion of its current development pathway, aiming to offer practical and actionable recommendations.

## Subjects and methods

### Basis for subject selection

This study employed a combination of questionnaire surveys and in-depth interviews, aiming to comprehensively explore perceptions, attitudes, usage habits, and acceptance of digital-intelligent healthcare across different demographic groups within society. The questionnaire survey was distributed extensively to respondents of varying ages, occupations, educational levels, and geographical distributions to ensure the breadth of the data.Participants were required to: ① possess basic electronic device proficiency to complete the questionnaire, and ② provide informed consent.

To further explore the views and practical experiences of different groups regarding digital -intelligent healthcare technologies, this study conducted in-depth interviews with nine representative participants. These included key groups such as medical university students, medical school faculty, elderly individuals, and clinical doctors. By integrating the perspectives of diverse groups, we were able to gain a more comprehensive understanding of the current status, challenges, and opportunities of digital-intelligent healthcare technologies, providing valuable guidance for the further development and application of these technologies.

### Methods

#### Literature review method

In the preliminary stage of the research, a systematic literature search and analysis focused on the three core topics of “high-quality development,” the “technology acceptance model (TAM),” and “digital-intelligent healthcare.” Using authoritative academic databases such as CNKI, PUBMED, and ProQuest, we extensively gathered various types of literature, including journal articles, dissertations (both doctoral and master’s), conference papers, policy guidance documents, and industry analysis reports. This work delineated the study’s scope and focus and laid a solid theoretical foundation for subsequent theoretical discussion and empirical analysis. Through meticulous sorting and synthesis of the literature, this study not only deepened the understanding of the core concepts of high-quality development but also systematically grasped the theoretical framework and application practices of the TAM while clarifying the latest progress and future trends in the field of digital-intelligent healthcare. During the literature review process, this study focused on comparing differing viewpoints across sources, striving to form a comprehensive understanding of the research topics, while keenly identifying gaps and shortcomings in existing research to provide targeted directions for subsequent investigations.

#### Questionnaire design

This study utilized a self-designed questionnaire as the data collection tool. The questionnaire design was based on the core constructs of the Technology Acceptance Model (TAM) and incorporated the specific characteristics and application scenarios of digital-intelligent healthcare to construct a multidimensional question system. The questionnaire consisted of two parts: the first part collected basic information, containing 11 items designed to gather respondents’ demographic characteristics and background information; the second part formed the main content, comprising 20 questions closely related to digital-intelligent healthcare, designed according to the TAM framework and covering key dimensions such as awareness, perceived usefulness, perceived ease of use, and behavioral intention, the specific structure of the questionnaire is shown in Table 2.

**Table 2:** Questionnaire structure

Section	Number of Items	Key Content	Question Type
Demographic Information	11	Age, occupation, education level, geographical region, etc.	Common type
Perceived Usefulness	2	Efficiency improvement, diagnostic accuracy, etc.	Likert-scale(1–5)
Perceived Ease of Use	2	Operation convenience, learning cost	
Subjective Norm	3	Perceived pressure from others’ expectations regarding behavior	
Behavioral Intention	3	Future use likelihood, recommendation willingness	
Attitude	3	Positive attitude toward technology	
Perceived Behavioral Control	3	Perception of difficulty and self-efficacy	
Technology Anxiety	1	Degree of technological distrust	
Social Influence	3	Degree of influence by the surrounding environment and individuals.	
**Total**	**31**		

In response to the development requirements from the perspective of high‑quality development, we contextualized the TAM within the domain of digital‑intelligent healthcare. Specifically, this study operationalizes perceived usefulness as the public’s recognition of digital‑intelligent healthcare in optimizing service experience and improving outcome accuracy, reflecting the goals of high quality and efficiency. Perceived ease of use is specified in terms of the convenience, low threshold, and time required to learn and use digital‑intelligent healthcare, linking it to service accessibility. Attitude and behavioral intention are measured directly through the public’s current level of positivity and satisfaction with the development of digital‑intelligent healthcare, as well as their likelihood of using or accepting such services in the future.

Furthermore, incorporating the characteristics of public decision-making, we introduced external variables such as subjective norms, perceived behavioral control, and social influence. These factors not only profoundly influence the public’s technology acceptance process but are also directly linked to the sustainability and realization of social value within the framework of high-quality development.

#### Interview outline design

**Fig. 1:**
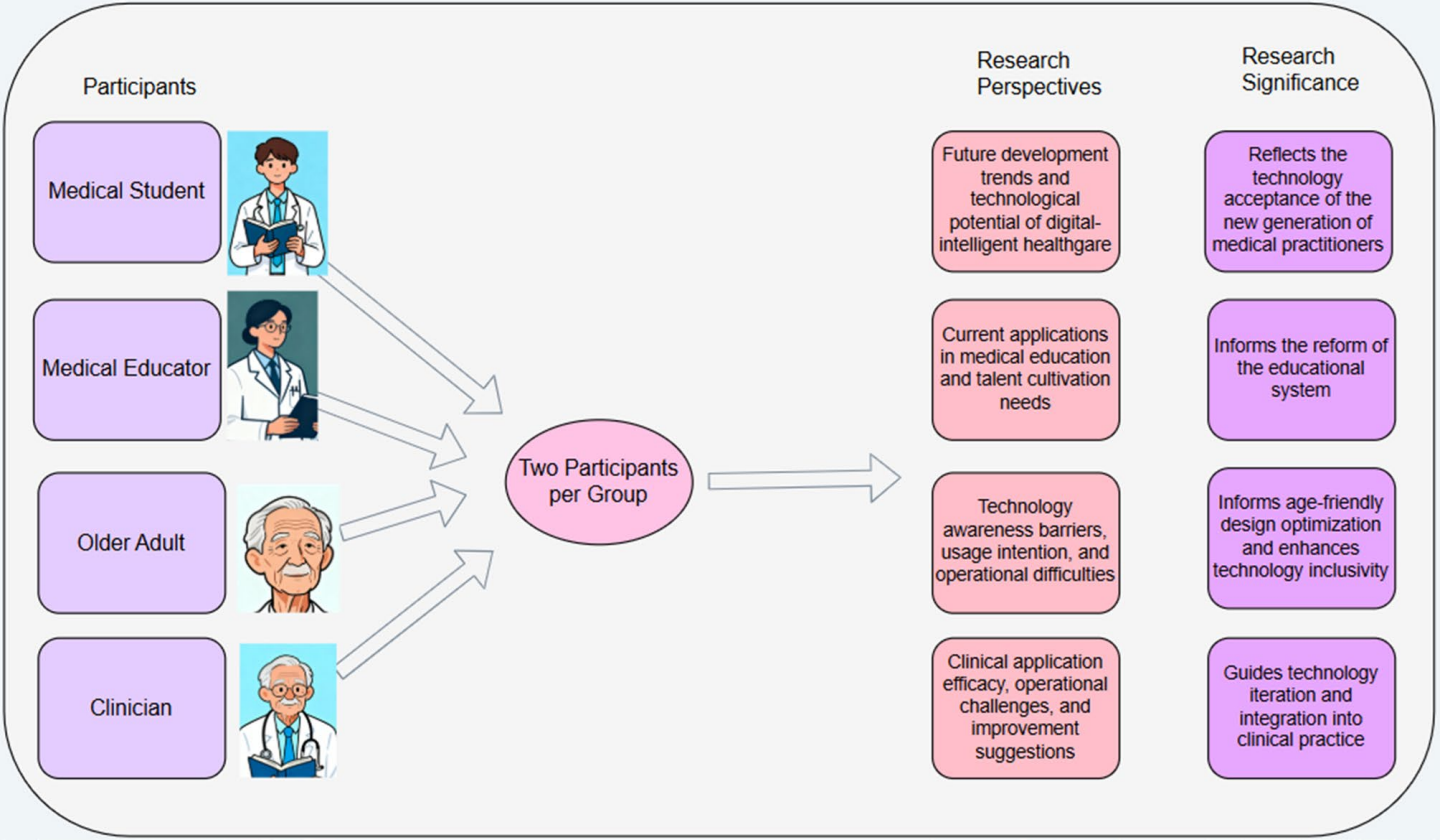
Interview framework and objectives

The interview outline designed for this study is closely aligned with the research objectives and the characteristics of the interviewees, aiming to delve into their cognition, attitudes, usage experiences, and improvement suggestions regarding digital-intelligent healthcare. The interview content encompasses multiple dimensions, including the advantages and limitations of digital-intelligent healthcare technologies, challenges and obstacles encountered during use, directions for improvement, and specific applications within teaching practices. To ensure the depth and accuracy of the interview content, tailored interview outlines were designed for different groups, such as healthcare professionals, patients, and educators, to foster an interview atmosphere that closely mirrors the actual contexts of the respondents, the interview procedure is illustrated in Fig. 1. Characteristics of the interviewees are presented in Table 3.

**Table 3:** Demographic profile of interviewees

Code	Gender	Age(Years)	Occupation	Major	Key Background	Region	Data Collection Method
A	Male	21	Undergraduate Student	Health Management	First-year student	Xinjiang	Face-to-face interview
B	Female	18	Undergraduate Student	Clinical Medicine	Third-year student	Jiangxi	Face-to-face interview
C	Female	45	Medical University Faculty	Nursing Science	Professor, Fellow of the American Academy of Nursing	Beijing	Video interview
D	Female	43	Medical University Faculty	Social Medicine	Professor	Beijing	Face-to-face interview
E	Male	52	Physician	Hepatology	Chief Physician, Tertiary A Hospital in a Tier-1 City	Beijing	Video interview
F	Female	40	Physician	Endocrinology	Chief Physician, Tertiary A Hospital in a Tier-2 City	Shandong	Video interview
G	Male	71	Retiree	Farmer	Hypertension patient	Beijing	Face-to-face interview
H	Female	69	Retiree	Enterprise	No underlying diseases	Beijing	Face-to-face interview

Occupation: Student/Physician/Retiree

Field/Specialty: Health Management/Clinical Medicine/Hepatology

### Data collection

This study disseminated questionnaires extensively through a combination of online and offline channels. The wide dissemination capability of social media platforms was utilized, while considering the relatively lower internet usage among older adults and individuals with lower educational levels. To ensure the diversity and representativeness of the survey sample, we conducted offline, paper-based questionnaire surveys targeting specific groups of elderly and less-educated individuals. Ultimately, a total of 456 questionnaire responses were collected. Recognizing that respondents completely unfamiliar with digital-intelligent healthcare might provide random or inaccurate answers when completing the scale, potentially compromising the comprehensiveness of the research conclusions and the overall data quality, we implemented rigorous logical screening and data cleaning procedures. This process yielded 340 valid questionnaires that were retained as the data sample for structural equation modeling (SEM) analysis. This meets the 10:1 ratio of sample size to free parameters recommended by Bentler & Chou [16] (1987), providing strong statistical power and stability for the estimation.

Offline interviewees were recruited through various methods, including posting recruitment posters, sending emails, and random street invitations. Nine voluntary participants were successfully invited. The interviews were conducted via a combination of in-depth personal interviews and a semistructured approach, carried out either face-to-face or via online meetings to ensure the depth and authenticity of the interview content. During the interviews, detailed records of the participants’ responses were kept. Systematic summarization and in-depth analysis were subsequently performed to extract both common themes and individual characteristics from the interview results.

### Quality control strategies

During the questionnaire design phase, this study strictly adhered to the principles of clarity, conciseness, specificity, and consistency. Core concepts such as perceived ease of use, perceived usefulness, and digital-intelligent healthcare were elucidated in layman’s terms to ensure that the questions were easily understandable. Upon completion of the initial draft, domain experts were invited to review the questionnaire, and its content was optimized on the basis of their feedback to guarantee professionalism and precision. Prior to formal distribution, a pilot test was conducted to identify and rectify ambiguous phrasing and logical flaws, significantly enhancing the questionnaire’s reliability and validity. In the data retrieval stage, invalid questionnaires were screened and removed one by one. The exclusion criteria were as follows: ① incomplete questionnaire responses, with omissions or errors; and ② logical inconsistencies within the questionnaire. Finally, statistical software such as IBM SPSS Statistics 27 and AMOS was used to conduct an in-depth analysis of the valid data, further verifying its quality.

**Fig. 2:**
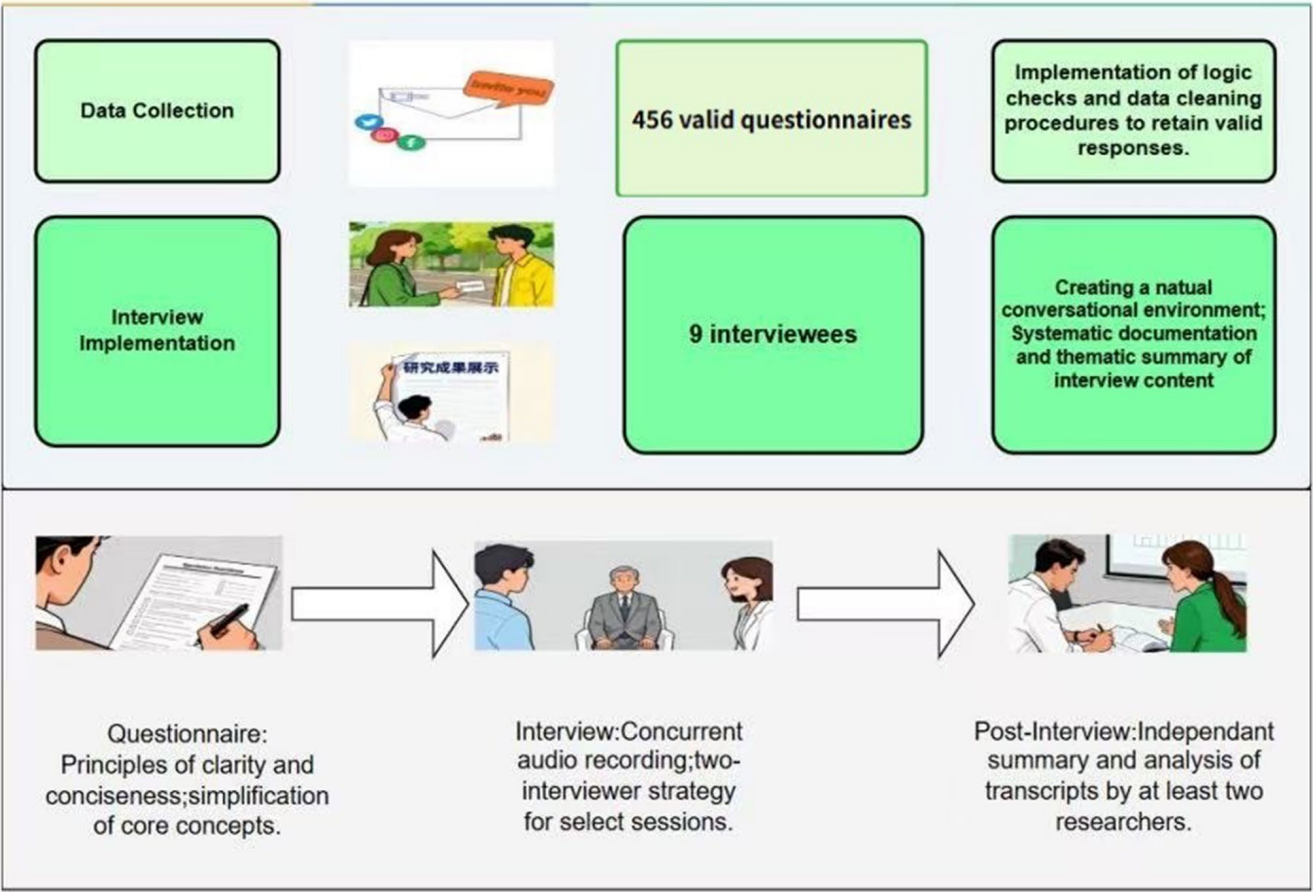
Data collection and quality control strategies

The interview outlines were tailored to different respondent groups while maintaining consistency in the core framework, ensuring the precision of the information gathered and its alignment with the research theme. During the interviews, a two-interviewer approach was adopted, and the entire process was audio-recorded. After the interviews, at least two individuals were assigned to summarize and analyse the recordings. Cross-verification was employed to prevent the omission of key information, thereby ensuring the richness and accuracy of the interview results, detailed information is provided in Fig. 2.

## Results

### Basic information

**Fig. 3:**
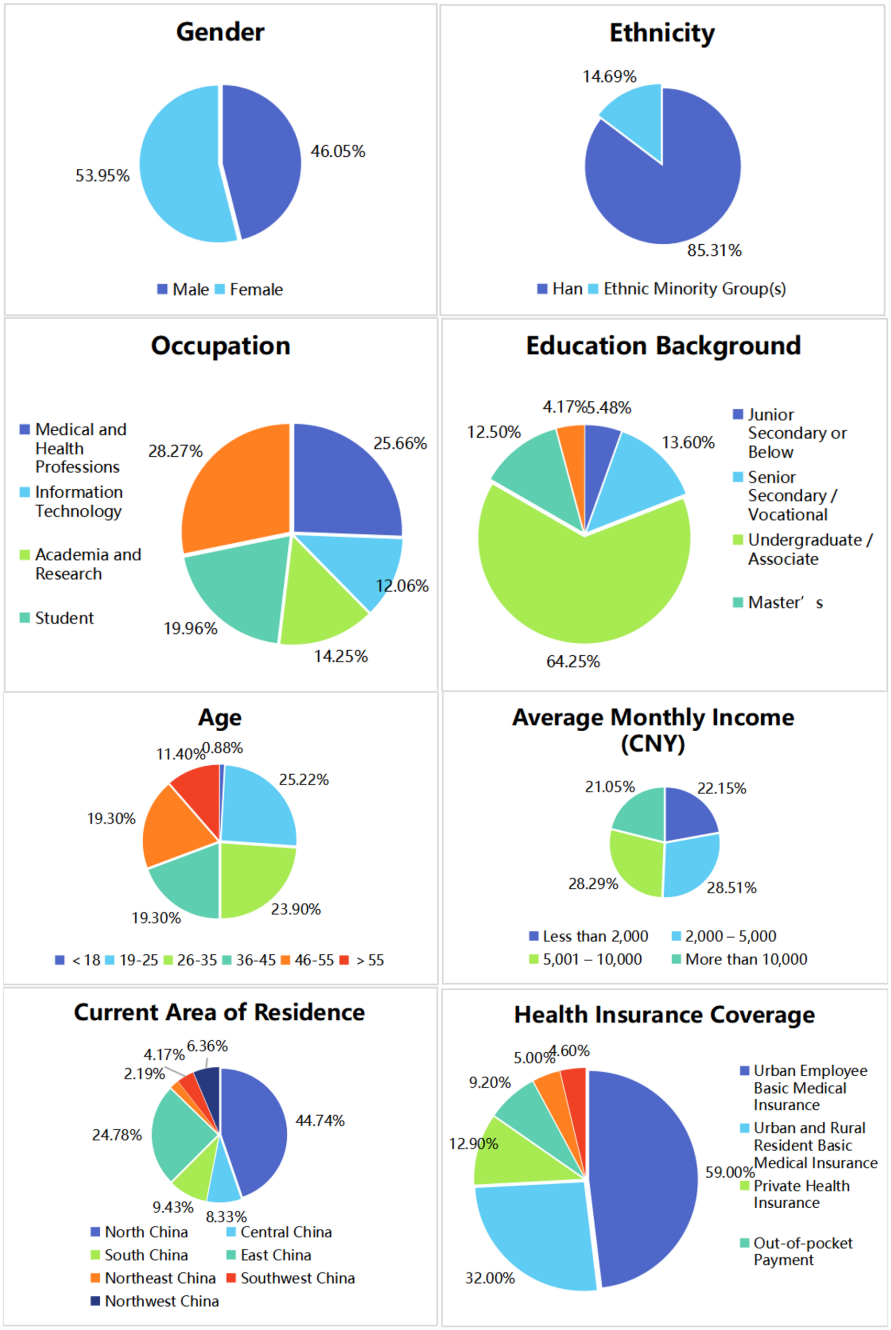

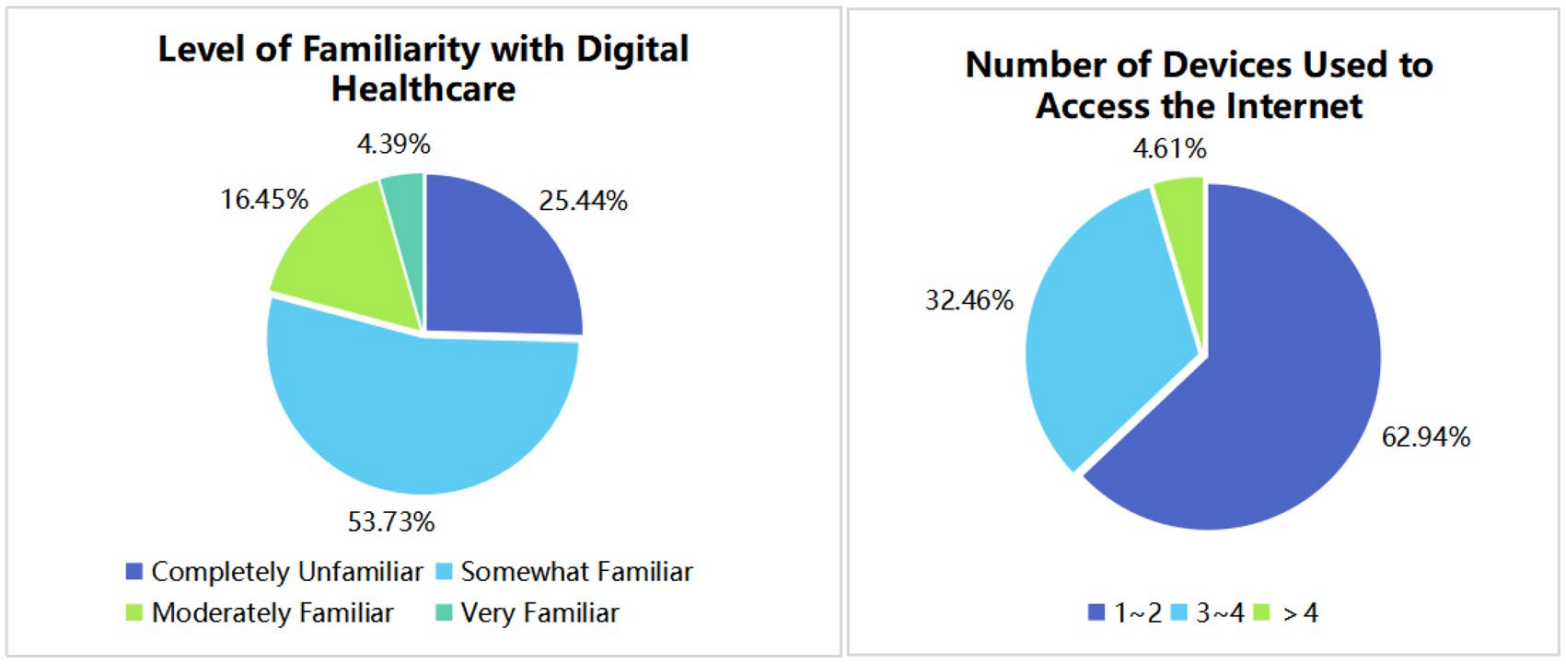
Demographic characteristics of respondents

A total of 456 valid questionnaires were collected, which is more than 10 times the number of scale items, indicating a moderate sample size. Among the respondents, men accounted for 46.05%, while women made up 53.95%. Regarding age distribution, approximately 26.10% of respondents were under 25 years old, 43.2% were aged 26–45, and around 30.7% were over 45, suggesting a relatively balanced age composition. In terms of educational background, the majority of respondents held a bachelor’s degree or an associate degree, accounting for 64.25%, followed by those with a high school diploma, secondary technical education, vocational high school education, or below, who constituted approximately 19.08%. Master’s degree holders and doctoral degree holders or above represented smaller proportions, at 12.50% and 4.17%, respectively. The respondents covered a wide range of occupational fields, primarily those related to the healthcare industry (25.66%) and students (19.96%), while other occupations, including retirees, accounted for approximately 28.07%. Regarding health insurance types, the majority of respondents were covered by the Basic Medical Insurance for Urban Workers and the Basic Medical Insurance for Urban and Rural Residents, accounting for about 59.00% and 32.00%, respectively. Furthermore, the questionnaire results also indicate that the current adoption rate of digital-intelligent healthcare is not high, with the majority of respondents reporting either no understanding (25.44%) or only a slight understanding (53.73%) of the concept. The detailed distribution of the results is presented in Fig. 3.

### Questionnaire reliability, validity, and model fit testing

The reliability and validity test results of the questionnaire showed that the KMO value was 0.932 (far exceeding the critical value of 0.5), and Cronbach’s alpha coefficient was 0.919 (exceeding the reliability standard of 0.7). These results indicate that the questionnaire possesses excellent construct validity and internal consistency reliability, demonstrating high data reliability.

The model fit test results showed that the chi-square value (CMIN) of the default model was 413.919 with 141 degrees of freedom, and the ratio of chi-square to degrees of freedom (CMIN/DF) was 2.936, indicating that the model fit was within an acceptable range. The RMR value was 0.062, the GFI value was 0.881, and the AGFI value was 0.840, all approaching the ideal values, suggesting a good goodness-of-fit for the model. Regarding the baseline comparison indices, the NFI value was 0.891, the RFI value was 0.868, the IFI value was 0.925, the TLI value was 0.909, and the CFI value was 0.925, all exceeding the threshold of 0.8, demonstrating a high degree of fit between the model and the data. The RMSEA value was 0.076, which is below the ideal value of 0.08. Overall, the model exhibited a good fit and could effectively explain the data. See Table 4.

**Table 4:** Model fit indices

Metric	CMIN/DF	RMR	GFI	AGFI	NFI	RFI	IFI	TLI	CFI	RMSEA
Fix Index	2.936	0.062	0.881	0.840	0.891	0.868	0.925	0.909	0.925	0.076
Recommended Value	<3	<0.08	>0.9	>0.8	>0.8	>0.8	>0.8	>0.8	>0.8	<0.8

### Path analysis results

**Fig. 4:**
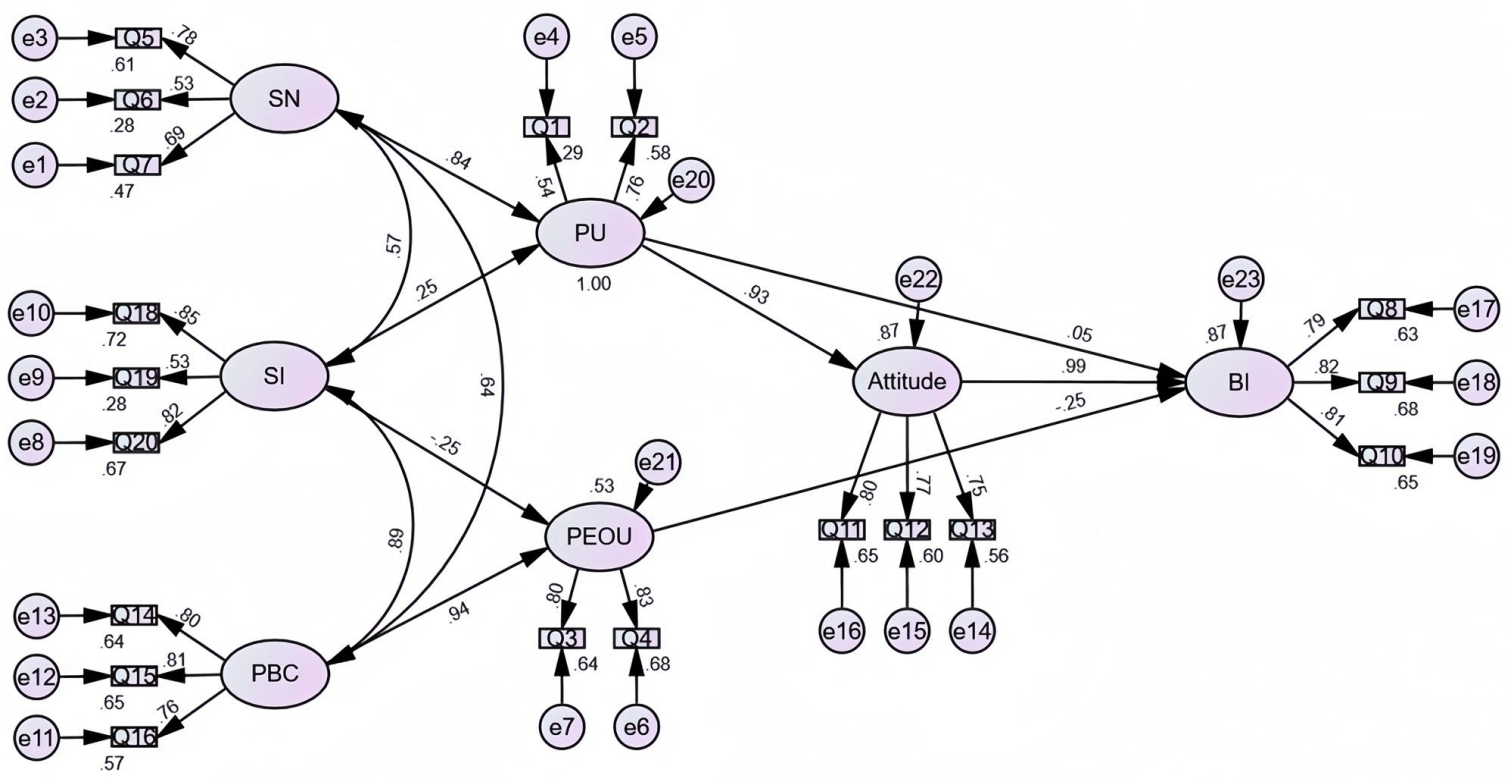
Path diagram of the structural equation modelling

As shown in the analysis results (see Table 5; Fig. 4), the standardized regression coefficient of subjective norms on perceived usefulness was 0.836, and it was statistically significant (*p* < 0.01), indicating that subjective norms have a significant positive influence on perceived usefulness. The standardized regression coefficient of social influence on perceived usefulness was 0.254, which was statistically significant (*p* < 0.01), suggesting that social influence has a positive effect on perceived usefulness. The standardized regression coefficient of social influence on perceived ease of use was − 0.246, which did not reach statistical significance (*p* > 0.05), indicating no statistical association between the two. The standardized regression coefficient of perceived behavioral control on perceived ease of use was 0.942, and it was statistically significant (*p* < 0.01), demonstrating that perceived behavioral control has a significant positive influence on perceived ease of use. Furthermore, the standardized regression coefficient of perceived usefulness on attitude was 0.934, and it was statistically significant (*p* < 0.01), showing that perceived usefulness has a significant positive influence on attitude. The standardized regression coefficient of attitude on behavioral intention was 0.991, and it was statistically significant (*p* < 0.01), indicating that attitude has a significant positive influence on behavioral intention. However, the standardized regression coefficient of perceived usefulness on behavioral intention was 0.047, which was not statistically significant (*p* > 0.05), suggesting no statistical association between these two variables. The standardized regression coefficient of perceived ease of use on behavioral intention was − 0.245, and it was statistically significant (*p* < 0.01), indicating that perceived ease of use has a negative influence on behavioral intention.

**Table 5:** Path coefficients

Hypothesis	Relationship	β	t-value	*P*-value	Supported
H1	Subjective Norm → Perceived Usefulness	0.836	8.090	<0.01	Yes
H2	Social Influence → Perceived Usefulness	0.254	3.704	<0.01	Yes
H3	Perceived Behavioral Control → Perceived Ease of Use	0.942	3.825	<0.01	Yes
H4	Perceived Usefulness → Attitude	0.934	9.150	<0.01	Yes
H5	Attitude → Behavioral Intention	0.991	3.687	<0.01	Yes
H6	Perceived Usefulness → Behavioral Intention	0.047	0.176	0.860	No
H7	Perceived Ease of Use → Behavioral Intention	-0.245	-4.186	<0.01	Yes
H8	Social Influence → Perceived Ease of Use	-0.246	-1.048	0.294	No

In view of the statistically non-significant direct effect of perceived usefulness on behavioral intention, we further conducted a mediating effect test. This study employed the Bootstrap method (*n* = 5000) to examine the mediating role of attitude in the relationship between perceived usefulness and behavioral intention. The results (as shown in Table 6) indicated that the total effect of perceived usefulness on behavioral intention was significant (β = 0.823, SE = 0.002, *p* < 0.01, 95% CI [0.546, 1.199]). After incorporating attitude into the model, the direct effect of perceived usefulness on behavioral intention was not significant (β = 0.040, SE = 0.660, *p* > 0.05, 95% CI [-1.676, 0.827]), while the indirect effect was significant (β = 0.784, SE = 0.591, *p* < 0.01, 95% CI [0.335, 2.636]), with the confidence interval excluding zero. Given that the direct effect was non-significant while the indirect effect was significant, this suggests that attitude plays a complete mediating role between perceived usefulness and behavioral intention.

**Table 6:** Mediating effect test

	Point estimate	SE	Bootstrap 5000 time 95% CI		*P*
			Lower	Upper	
Indirect Effect	0.784	0.591	0.335	2.636	<0.01
Direct Effect	0.040	0.660	-1.676	0.827	>0.05
Total Effect	0.823	0.002	0.546	1.199	<0.01

The finding that attitude plays a complete mediating role between perceived usefulness and behavioral intention deepens our understanding of the adoption mechanism of digital-intelligent healthcare. In contrast to the assumptions of the classic TAM model (where PU directly drives BI), the decision-making path for users appears more indirect in the context of digital-intelligent healthcare, which involves health management. The establishment of complete mediation also suggests that current users of digital-intelligent healthcare may still be in a technology adaptation period, where their perception of ‘usefulness’ has not yet been automatically translated into action, necessitating the bridging role of attitude. This finding provides a precise target for subsequent interventions: enhancing user attitudes (e.g., through experiential training and improving the user-friendliness of human-computer interaction) is more effective in boosting the adoption rate of digital-intelligent healthcare than simply emphasizing its usefulness.

Social influence and subjective norms regarding digital-intelligent healthcare exert a positive effect on perceived usefulness. This finding indicates that the tangible efficacy of the technology (e.g., remote consultations effectively reduce waiting times, AI-assisted diagnosis improves accuracy rates) allows people to intuitively appreciate the conveniences offered by digital-intelligent healthcare, thereby facilitating the formation of positive word-of-mouth through interpersonal communication. Policy drivers further reinforce public recognition of the value of technology. For example, the key tasks for deepening the Medical and Health System Reform in 2024 explicitly integrated digital-intelligent healthcare into the service system, encouraging the public to translate the positive attitude of “public recognition” into concrete social behaviors such as “family recommendations” and “doctor guidance.”

Research by Rodriguez et al. in the United States [17] on the Spanish-speaking population provides corroborating evidence for this conclusion. Their study revealed that the usage rate of technologies such as telemedicine and digital health among the U.S. Spanish-speaking population is significantly lower than that among the English-speaking population because of factors such as lower literacy levels and insufficient digital and intelligent literacy. However, proactive guidance from healthcare providers can markedly increase their usage of digital-intelligent healthcare tools. Owing to the nature of their roles, healthcare professionals and patients frequently interact with technologies related to digital-intelligent healthcare. Their positive attitudes are amplified by their professional status, making their perceptions of the usefulness and ease of use of digital-intelligent healthcare products more persuasive.

Perceived behavioral control positively influences perceived ease of use. The interviews targeting the elderly population in this study revealed that operational complexity (e.g., multilevel menus, font size being too small) directly inhibits their willingness to use the technology. Conversely, optimizing interactive design (e.g., adding larger buttons, voice guidance) can significantly increase their likelihood of recommending the technology to their peers. Research by Sun [18] et al. on the usage intention of smart medical devices among older Chinese adults revealed that “technical interactivity” completely mediates the relationship between “perceived usefulness” and “usage intention.” This suggests that enhancing the operability of digital-intelligent healthcare products, making it easier for users to interact with them, can effectively lower the cognitive barrier to using these products, thereby promoting the penetration of technology into groups with lower levels of cognitive familiarity.

Moreover, the questionnaire survey data revealed that only 16.54% of the respondents reported being “relatively familiar” or “very familiar” with digital-intelligent healthcare. This low level of prevalence further underscores the necessity of improving the ease of use of existing products and incorporating age-friendly design into future products.

After patients use or experience services such as intelligent appointment scheduling and health monitoring, they tangibly feel how digitally intelligent healthcare products streamline medical procedures and increase convenience in their lives. This reinforces their recognition of the technology’s usefulness and ease of use, ultimately forming a positive feedback loop of “usage → positive attitude → continued usage.” Among them, younger demographic groups (19–25 years old, comprising 38.98% of the sample), leveraging their higher digital literacy and frequency of smart device use, are more likely to reinforce this loop through high-frequency usage. Owing to their occupational characteristics, healthcare professionals have become key nodes in the diffusion of this technology.

Additionally, we note that the results of the study indicate a negative impact of perceived ease of use on behavioral intention. This somewhat unexpected finding should not be overinterpreted and can be explained from two perspectives. On one hand, in complex medical decision-making contexts, if a technology excessively prioritizes interface simplification or process automation, it may lead professional users (such as physicians) to perceive it as inadequately capturing the complexity of clinical work, thereby casting doubt on the reliability of its outputs. A 2025 study on clinical decision support system alerts [19] corroborates the plausibility of this observation. On the other hand, the rapid pace of technological advancement has fostered public distrust toward the security of technologies or services. Within the surveyed sample, approximately 43.82% of individuals exhibited signs of technology anxiety—a concern also repeatedly raised during interviews. Strong technology anxiety can act as a significant psychological barrier. In such cases, even if individuals objectively consider a system easy to learn, anxiety may dominate their decision-making, leading to avoidance behaviors. Consequently, the relationship between perceived ease of use and behavioral intention may appear negative or insignificant. A 2025 study on radar-based health monitoring technology [20] explicitly highlights that technology anxiety can weaken the influence of core TAM constructs, such as perceived ease of use, on usage intention.

### Interview results

Through interviews with representatives from diverse demographics, we have identified some prevailing issues in the current development of digital-intelligent healthcare, which has informed our subsequent discussions and the formulation of recommendations.

Regarding the current state of digital-intelligent healthcare usage, although it has greatly contributed to the convenience of medical services, there are certain shortcomings in areas such as age-friendly adaptation, technical accuracy, and resource allocation, which hinder its further popularization and development. Among these, the lack of adequate age-friendly adaptation was mentioned frequently. For example, two doctors both noted that in their work, they observed that elderly individuals generally have insufficient digital literacy, and operating digital medical devices tends to be cumbersome for older patients—especially for those with limited education at the grassroots level (see Supplementary File 6-Interviewee E; Supplementary File 7-Interviewee F). Some interviewed seniors pointed out that during online consultations, inconsistent results were sometimes obtained after repeated inquiries, making it difficult to receive reliable reference outcomes (see Supplementary File 8-Interviewee G; Supplementary File 9-Interviewee H). Beyond this, limited access to equipment and technology at the grassroots level is also a key issue. The interviewed doctors emphasized that under the tiered healthcare policy, strengthening resource allocation in community hospitals at the primary level could enhance remote collaborative consultations between hospitals of different tiers, thereby providing more efficient services for patients (see Supplementary File 6-Interviewee E; Supplementary File 7-Interviewee F).

Regarding expectations for digital-intelligent healthcare, the view that its rapid advancement is an inevitable trend appears to have become mainstream. Interviewees generally expressed high willingness to adopt and promote digital-intelligent healthcare in the future, maintaining an optimistic outlook overall. Although some teachers and doctors noted that the widespread use of digital-intelligent technology may, to some extent, encroach on healthcare professionals’ personal time through increased online consultations, and that data security remains a significant concern, the consensus is that the benefits of popularizing digital-intelligent healthcare outweigh the drawbacks. Meanwhile, all respondents emphasized the importance of enhancing digital literacy among medical practitioners. Teachers and students alike showed considerable interest in integrating intelligent technology into medical education, which lays a solid and optimistic foundation for the future development of digital-intelligent healthcare (see Supplementary File 2-Interviewee A; Supplementary File 3-Interviewee B; Supplementary File 4-Interviewee C; Supplementary File 5-Interviewee D).

## Discussion and suggestions

### Advantages and disadvantages of the TAM in digital-intelligent healthcare research

As a classic theoretical framework for technology acceptance research, the technology acceptance model has unique advantages in digital-intelligent healthcare studies. First, the TAM has a simple structure, and its core variables are easy to measure, enabling rapid assessment of user acceptance of digital-intelligent healthcare technologies. Second, the TAM possesses high predictive ability, providing valuable references for medical institutions to optimize digital-intelligent healthcare systems.

However, the TAM also has certain limitations. First, it assumes that user attitudes are static, whereas in reality, user acceptance of digital-intelligent healthcare dynamically changes with increasing usage experience. Second, the model’s general structure may not fully capture context-specific relationships. For instance, in this study, the path from “Perceived Usefulness” to “Perceived Behavioral Control” was not statistically significant (β = -0.003, *p* > 0.05). This suggests that, in the context of digital-intelligent healthcare, believing a system to be useful does not directly enhance one’s confidence in being able to use it. This discrepancy may indicate the influence of other mediating or moderating variables (e.g., age, digital literacy, or training support) that were not included in the current model. Future research could therefore integrate additional constructs or employ dynamic analytical frameworks to further refine the TAM’s applicability and explanatory power in the field of digital-intelligent healthcare.

### Key determinants influencing the use of digital-intelligent healthcare

The data results of this study reveal a clear transmission mechanism: subjective norms serve as the most significant antecedent influencing perceived usefulness (β = 0.836, *p* < 0.01), while perceived usefulness acts as the most direct and dominant variable influencing attitude (β = 0.934, *p* < 0.01). Subsequently, attitude exerts a direct impact on behavioral intention (β = 0.991, *p* < 0.01). This finding indicates that although an individual’s behavioral intention is ultimately dominated by their judgment of the product’s usefulness, this judgment itself is strongly influenced by the opinions of others within the social environment. Therefore, subjective norms fundamentally drive behavioral intention through perceived usefulness.

The respondents in this study encompassed individuals across different age groups and varying levels of digital literacy. Among them, middle-aged and young adults (aged 18–45) accounted for 68.42% of the sample, while middle-aged and older adults (aged 46 and above) represented approximately 30.70%. As core potential users of digital-intelligent healthcare, the perceived usefulness among middle-aged and young adults is easily influenced by external opinions from peers, healthcare professionals, and other sources (subjective norms). Research by Shen Yun et al. (2025) on the willingness of older adults to use smart wellness devices also indicated that subjective norms have a significant positive impact on their adoption intention [21]. This finding helps explain the high path coefficient of subjective norms on perceived usefulness observed in the present study. Although the sources of subjective norms may differ across age groups, they consistently exert a significant positive influence on the perceived usefulness of digital-intelligent healthcare.

Additionally, the findings of this study further validate and extend the integrated application of the Technology Acceptance Model (TAM) and the Theory of Planned Behavior (TPB) in the field of digital-intelligent healthcare. However, it is worth noting that the conclusions of this study differ from those presented in “Analysis of Factors Influencing User Acceptance Behavior of Mobile Health Applications” [22], which stated that “subjective norms do not affect users’ perceived usefulness.” This discrepancy may be attributed to the fact that the cited study focused on a single mobile health application, whereas digital-intelligent healthcare constitutes a comprehensive healthcare service model. In this context, individuals’ judgments of its usefulness are more likely to rely on external social feedback and subjective norms. This also highlights the moderating effect of research context on variable relationships, providing a reference for subsequent studies in differentiated scenarios.

### Conclusions

This study employs a mixed-methods approach, integrating questionnaires and in-depth interviews within the Technology Acceptance Model (TAM) framework and a high-quality development perspective, to systematically investigate the influencing mechanisms of the public’s willingness to accept digital-intelligent healthcare. The findings reveal that public acceptance of digital-intelligent healthcare is not driven merely by technological functionality but is a complex process modulated by multiple factors, including social cognition, psychological experience, and organizational environment. The main conclusions are as follows.

#### Social-psychological mechanisms: the conductive pathway from normative cognition to behavioral intention

##### Perceived usefulness as the core cognitive mediator of acceptance behavior

Perceived usefulness not only directly and positively influences behavioral intention (β = 0.462, *p* < 0.05) but also fully mediates the effect of attitude on usage intention. This finding confirms the robustness of TAM in the context of healthcare digitization while revealing a deeper transmission mechanism: the public’s judgment of “usefulness” is strongly shaped by social norms. In this study, the impact coefficient of subjective norms on perceived usefulness was as high as 0.783 (*p* < 0.01), indicating that social information such as physician recommendations, policy guidance, and community word-of-mouth acts as both a “filter” and an “amplifier” in the formation of perceived technological value. This finding aligns with the forefront of global AI-in-healthcare research, which posits that public and healthcare professionals’ awareness and attitudes are key socio-psychological filters for technology adoption [23]. Therefore, promoting digital-intelligent healthcare must focus not only on technical performance but also on constructing a positive social cognitive environment.

##### Perceived ease of use exhibits a “trust threshold” effect on behavioral intention

Contrary to the classic TAM expectation, this study found a significant negative impact of perceived ease of use on behavioral intention (β = -0.405, *p* < 0.01). This “counter-intuitive” result reveals that in high-trust, high-expertise medical fields, the sole pursuit of operational simplicity may trigger user skepticism regarding the technology’s reliability. Qualitative analysis of online reviews for telemedicine by Binsar et al. (2025) similarly found that negative sentiments often centered on issues such as “lack of system transparency,” “anxiety about privacy risks,” and “rigid human-computer interaction” [24], suggesting that Technology anxiety stems not only from operational difficulty but is deeply rooted in fundamental doubts about system trustworthiness. This further indicates that the application of AI in healthcare must be understood as a “socio-technical phenomenon”, requiring the integration of transparency, explainability, and ethical considerations into technological design [23].

#### Organizational and environmental factors: the criticality of capacity building and ecosystem synergy

##### Digital leadership and ICT literacy as cornerstones of organizational adoption

Perceived behavioral control exhibited a strong positive effect on perceived ease of use (β = 0.734, *p* < 0.01), indicating that enhancing users’ operational confidence and sense of control is an effective way to lower the barrier to use. Further research by Binsar et al. (2024) on hospitals’ digital adoption capabilities points out that digital leadership and staff ICT literacy are the two pillars for an organization’s successful digital transformation [25]. Leaders must not only possess technological vision but also translate external norms (e.g., policy requirements) into internal drivers through institutional design, resource allocation, and cultural shaping. Furthermore, the digital literacy of healthcare practitioners—encompassing information screening, ethical judgment, and system operation skills—directly impacts the safety and effectiveness of technology implementation [25]. This conclusion is supported by international management research, which similarly emphasizes that hospital managers must invest in technology integration, personnel training, and change management to overcome adoption barriers and unleash AI’s potential value [23].

##### Environmental dynamism demands synchronization between technology promotion and policy rhythm

Both interview and questionnaire data from this study indicate that external events and policies, such as the COVID-19 pandemic and China’s “14th Five-Year Plan for Digital Economy Development,” significantly accelerated public awareness and trial use of digital-intelligent healthcare. Binsar et al. (2024) define such external pressures and opportunities as “environmental dynamism” and stress that successful digital adoption must maintain strategic alignment with policy evolution and social change [25]. This implies that technology promotion should not be an isolated “product launch” but rather a “system adaptation” embedded within the macro-process of healthcare reform. From the perspective of the Knowledge-Based View (KBV), this synergy is essentially a process of interdisciplinary knowledge transfer and innovation diffusion [23], requiring policymakers, technology developers, and healthcare providers to establish ongoing knowledge dialogue and collaboration mechanisms.

#### From public feedback to patient-centeredness: constructing the experience loop

##### Online public opinion as a “real-time sensor” for gauging public sentiment

Analysis of massive online reviews by Binsar et al. (2025) revealed that public discussion of telemedicine focuses on three key dimensions: service accessibility, cost transparency, and personalized experience [24]. This aligns closely with concerns raised in the interviews of this study, such as “age-friendly design,” “fee clarity,” and “operational guidance.” This demonstrates that social media and online reviews have become critical arenas for the generation and diffusion of contemporary “social norms,” where positive word-of-mouth can significantly enhance technological credibility, while the spread of negative experiences can rapidly erode adoption willingness [24]. Therefore, monitoring and responding to online public opinion should become a routine task for digital-intelligent healthcare operators.

##### “Patient-centeredness” is not a slogan but a design principle for the experience loop

This study found that younger demographics (19–25 years old), due to higher digital literacy and more frequent use, are more likely to form a positive cycle of “use → identification → recommendation.” In contrast, older groups pay more attention to features such as “clear interface,” “voice assistance,” and “family support linkage.” The integrated framework proposed by Binsar et al. (2025) further suggests that the ultimate goal of innovation should be to achieve a virtuous cycle of “challenge-benefit-innovation”—that is, transforming user pain points into service highlights by precisely responding to patient feedback (e.g., reducing waiting times, optimizing appointment processes), thereby constructing a continuously improving patient experience loop [24]. This philosophy is highly consistent with the “Social and Ethical Aspects” cluster in AI healthcare research, which emphasizes that community engagement, equitable access, and communication effectiveness are central to responsible innovation [23].

#### Policy implications and directions for future research

Based on the above findings, we propose the following hierarchical and actionable policy recommendations, which resonate with the strategic consensus of the international academic community on the development of AI in healthcare [23]. The specific details are shown in Table 7.

**Table 7:** Policy recommendations

Target Dimension	Core Strategy	Specific Measures
Individual Cognitive Level	Alleviate Technology anxiety and enhance sense of control	1.Launch community-based “Digital Health Navigator” training programs to provide face-to-face assistance. 2. Establish “Digital-Intelligent Healthcare Experience Corners” in public hospitals to offer risk-free trial environments. 3. Develop “Transparency Mode” features to show users the logical basis of AI-assisted decision-making, addressing concerns about the “black box.”
Organizational Promotion Level	Cultivate digital leadership and staff literacy	1. Incorporate “Digital Leadership” into the performance evaluation metrics for directors of public hospitals. 2. Collaborate with universities to offer “Healthcare ICT Literacy” micro-degree programs, counting towards continuing education credits. 3. Establish “Hospital-Enterprise-University” digital healthcare talent co-development bases to strengthen capabilities in change management and technological operations.
Ecosystem Synergy Level	Build a policy-technology-society collaborative network	1. Drawing on international calls [23], accelerate the formulation of standards for age-friendly digital-intelligent healthcare products, algorithm transparency norms, and data security guidelines, and incorporate them into medical device registration review requirements. 2. Designate “Digital Healthcare Social Experiment Zones” to encourage enterprises, hospitals, and communities to co-create service models, validating technology acceptability and effectiveness through community-based approaches. 3. Establish cross-departmental (health, medical insurance, industry and information technology) data sharing and operational linkage mechanisms to overcome the dilemmas of “data silos” and “policy fragmentation.”

### Relationships between digital-intelligent healthcare and offline healthcare

Digital-intelligent healthcare and offline healthcare are not substitutive but rather achieve optimal allocation of medical resources through functional complementarity and system integration. In terms of service scenarios, digital-intelligent healthcare overcomes spatiotemporal limitations via remote consultation platforms, effectively alleviating the uneven geographical distribution of medical resources. Offline healthcare, meanwhile, remains irreplaceable in services requiring physical interaction and high complexity, with advantages manifested in the necessity of physical contact, device dependency, and the building of doctor–patient trust. Future research should focus on defining responsibilities and rights in human‒computer collaboration and constructing ethical frameworks, promoting the development of medical service systems toward greater efficiency and fairness.

In the future, digital-intelligent healthcare should integrate multimodal medical data and continuously optimize diagnostic models. Interviewee C suggested that digital-intelligent healthcare should develop towards greater refinement and individualization. For example, using smaller, smarter probes is better suited to the human body during gastroscopy to reduce discomfort during the examination (see Supplementary File 4-Interviewee C). Litjens et al. emphasized the powerful role of multimodal data fusion in enhancing the precision and personalization of medical services [26]. Digital-intelligent healthcare should deeply integrate multimodal medical data, introduce cutting-edge artificial intelligence algorithms to continuously optimize diagnostic models, and tailor ultrafined, highly individualized, and highly accurate dynamic diagnosis and treatment plans on the basis of patient health trajectories and lifestyle habits.

### Study limitations

This study has two limitations. First, there is potential sample selection bias. Influenced by the online dissemination channels, the questionnaire distribution was primarily concentrated in specific groups, and urban residents accounted for a relatively high proportion, while the needs of rural areas were not fully reflected, leading to insufficient generalizability of the research results. This study employed a convenience sampling method, which is subject to convenience sampling bias. Therefore, the results should be generalized to the population with caution. Additionally, the self-reported nature of the survey carries inherent risks of social desirability bias, casual responses, or comprehension inaccuracies.

To enhance the robustness and representativeness of future research, we recommend: ①employing stratified random sampling to ensure a balanced representation across key demographics such as age, education level, urban-rural residence, and occupational field; ②significantly increasing the sample size to improve statistical power and stability, while considering the inclusion of mediating or moderating variables in the model to explore more deeply the pathways influencing public attitudes and intentions toward digital-intelligent healthcare.

## Supplementary Information

Below is the link to the electronic supplementary material.

Supplementary Material 1

Supplementary Material 2

Supplementary Material 3

Supplementary Material 4

Supplementary Material 5

Supplementary Material 6

Supplementary Material 7

Supplementary Material 8

Supplementary Material 9

## Data Availability

The data supporting this studyʼs findings are available upon reasonable request from the corresponding author.

## Abbreviations

AI: Artificial intelligence
AGFI: Adjusted goodness-of-fit index
BI: Behavioral intention
CFI: Comparative fit index
CMIN: Chi-square value
DF: Degrees of freedom
GFI: Goodness-of-fix index
IFI: Incremental fit index
NFI: Normed fix index
PBC: Perceived behavioral control
PEOU: Perceived ease of use
PU: Perceived usefulness
RFI: Relative fix index
RMR: Root mean square residual
RMSEA: Root mean squared error of approximation
SEM: Structural equation modelling
SI: Social influence
SN: Subjective norm
TAM: Technology acceptance model
TLI: Tucker-Lewis index

## References

[1] Jiang QQ, Zhang ZH, Xiong JJ, et al. Research on patients’ adoption intention to internet+nursing service based on the technology acceptance model and theory of planned behavior theory. Nurs J Chin PLA. 2022;39(2):42–5.

[2] Jiao YL, Jin Y, Geng YM, et al. Analysis of influencing factors of digital transformation of outpatient service in shanghai municipal hospitals based on technology acceptance model. Chin Hosp Manage. 2024;44(8):46–9.

[3] Ma CC, Zhu HW, Zhang WY, et al. Construction and practice of the optimized management pathway for integrated health care of two chronic diseases at the primary level empowered by digital intelligence. Chin Rural Health. 2024;16(2).

[4] Guo XY. Galloping on the new track of smart healthcare: information technology innovation: forging the foundation of high-quality medical services. In: Proceedings of the 17th Annual Conference of China Hospital CEO. 2023: 85–87.

[5] Li CY, Shi L. Discussion on key elements and implementation path in the digital transformation of hospitals. Chin Hosp Manage. 2023;43(1):53–6.

[6] Lyu YB, Yu L, Li HT, et al. Analysis of high-quality development path of hospitals based on digital intelligence three-dimensional system. Data Technol Appl. 2023;41(10):92–4.

[7] Pan J, Zhang TF, Zhang YM, et al. Digital intelligence drives the high-quality development of the healthcare service system: development mechanisms and implementation pathway. J Sichuan Univ (Medical Science). 2024;55(5):1055–62. 10.12182/20240960401 PMC11536252 39507985

[8] Ruan HY, Wu TX. Exploration and application of information management in hospital rehabilitation nursing under the background of smart healthcare. Inform China. 2025;(2):209–11.

[9] Zhu CE, Zheng HL, Zhu HY, et al. Study on the influence mechanism of the use of hospital intelligent medical system on patients’ satisfaction: based on the perspective of technology acceptance model. Chin Hosp Manage. 2019;39(10):61–4.

[10] Liu JYW, Sorwar G, Rahman MS, Hoque MR. The role of trust and habit in the adoption of mHealth by older adults in Hong Kong: a healthcare technology service acceptance (HTSA) model. BMC Geriatr. 2023;23(1):73. 10.1186/s12877-023-03779-4. PMID: 36737712; PMCID: PMC9898708. 36737712 10.1186/s12877-023-03779-4 PMC9898708

[11] Li J, Ma Q, Chan AH, Man SS. Health monitoring through wearable technologies for older adults: Smart wearables acceptance model. Appl Ergon. 2019;75:162–9. 10.1016/j.apergo.2018.10.006. Epub 2018 Oct 25. PMID: 30509522. 30509522 10.1016/j.apergo.2018.10.006

[12] Luo J, Zhang K, Huang Q, Jiang S, Pan Y. From acceptance to dependence: exploring influences of smart healthcare on continuous use intention of mobile health services among older adults with chronic illnesses in China. Behav Sci (Basel). 2024;15(1):19. 10.3390/bs15010019. PMID: 39851823; PMCID: PMC11762675. 39851823 10.3390/bs15010019 PMC11762675

[13] Yang HJ, Lee JH, Lee W. Factors influencing health care technology acceptance in older adults based on the technology acceptance model and the unified theory of acceptance and use of technology: meta-analysis. J Med internet Res. 2025;27:e65269. 10.2196/65269. PMID: 40153796; PMCID: PMC11992498. 40153796 10.2196/65269 PMC11992498

[14] Joo OE, Ha YK. Factors affecting the intention to use smartmonitor-based mobile health in middle-aged in patients applying the technology acceptance model II. J Korean Acad Nurs. 2024; 54(4):620–632. Korean. 10.4040/jkan.24091. PMID: 39663624. 10.4040/jkan.24091 39663624

[15] Chen CH, Lee WI. Exploring nurses’ behavioural intention to adopt AI technology: the perspectives of social influence, perceived job stress and human–machine trust. J Adv Nurs. 2025; 81(7):3739–3752. 10.1111/jan.16495. PMID: 39340769. 10.1111/jan.16495 39340769

[16] Bentler PM, Chou CP. Practical issues in structural modeling. Sociol Method Res. 1987;16(1):78–117. 10.1177/0049124187016001004.

[17] Higashi RT, Thakur B, Repasky EC, et al. Digital health technology use among Spanish speakers in the US: A scoping review. JAMA Netw Open. 2025;8(5):e2510386. 10.1001/jamanetworkopen.2025.10386. PMID: 40372754; PMCID: PMC12082372. 40372754 10.1001/jamanetworkopen.2025.10386 PMC12082372

[18] Sun S, Jiang L, Zhou Y. Associations between perceived usefulness and willingness to use smart healthcare devices among Chinese older adults: The multiple mediating effect of technology interactivity and technology anxiety. Digit Health. 2024;10:20552076241254194. 10.1177/20552076241254194. PMID: 38812850; PMCID: PMC11135081. 38812850 10.1177/20552076241254194 PMC11135081

[19] Chien SC, Chien CH, Chen CY, Chien PH, Hsu CK, Yang HC, Li YC. Using technology acceptance model to explore physicians’ perspectives of clinical decision support system alerts. BMJ Health Care Inf. 2025;32(1):e101128. 10.1136/bmjhci-2024-101128. PMID: 40578848; PMCID: PMC12207143. 10.1136/bmjhci-2024-101128 PMC12207143 40578848

[20] Ho DH, Lee BH, Jeon B, Kim HS. Psychological Barriers and Perceptual Drivers of Sensor-Based Smart Health Technology Adoption. Sens (Basel). 2025;25(22):7029. 10.3390/s25227029. PMID: 41305236; PMCID: PMC12656334. 10.3390/s25227029 PMC12656334 41305236

[21] Shen Y, Li Q. Willingness to use smart wellness devices among older adults in the digital age and its influencing factors—an empirical study based on the deconstructed theory of planned behavior. Sci Res Aging 2025,13(2):33–47. 10.3969/j.issn.2095-5898.2025.02.003.

[22] Shao T, Wang X. Analysis of factors influencing user acceptance behavior of mobile health applications. J Xiamen Univ Technol. 2017;25(4):26–32.

[23] Hamsal M, Binsar F. Toward a new era of healthcare services: the role of artificial intelligence in shaping tomorrow’s landscape. Cogent Bus Manage. 2025;12(1):2566441.

[24] Binsar F, Mursitama TN, Hamsal M, Rahim RK. The role of digital adoption capability on hospital performance in Indonesia moderated by environmental dynamism. J Health Organ Manag. 2025;39(1):1–21. 41126338 10.1108/JHOM-04-2024-0130

[25] Binsar F, Mursitama TN, Hamsal M, Rahim RK. Determinants of digital adoption capability for service performance in Indonesian hospitals: a conceptual model. J Syst Manage Sci. 2024;14(2):188–213.

[26] Litjens G, Kooi T, Bejnordi BE, et al. A survey on deep learning in medical image analysis. Med Image Anal. 2017;42:60–88. 10.1016/j.media.2017.07.005. Epub 2017 Jul 26. PMID: 28778026. 28778026 10.1016/j.media.2017.07.005

