# Supplementary material for: Research on the implementation path of digital-intelligent healthcare based on the TAM model from the perspective of high-quality development
Source: BMC Health Serv Res. 2026 Mar 27;26:646. doi: 10.1186/s12913-026-14433-1 (PMC13151098; doi:10.1186/s12913-026-14433-1)
Supplement: Supplementary file 1 — Supplementary Material 1 [file 12913_2026_14433_MOESM1_ESM.docx]

Questionnaire for Research on the Implementation Path of Digital- Intelligent Healthcare

**Part Ⅰ Basic Information**

1.What is your gender?

[ ] Male
[ ] Female

2.What is your age range?

[ ] 18 or below
[ ] 19-25
[ ] 26-35
[ ] 36-45
[ ] 46-55
[ ] 56 or above

3.What is your highest level of education?

[ ] Junior high school or below
[ ] High school / Vocational school / Technical secondary school
[ ] Bachelor's degree / Associate degree
[ ] Master's degree
[ ] Doctoral degree or above

4.What is your current professional field?

[ ] Healthcare
[ ] Information Technology
[ ] Education / Research
[ ] Student
[ ] Other: [Please specify in this area]

5. What is your current region of residence in China?
[ ] North China
[ ] Central China
[ ] South China
[ ] East China
[ ] Northeast China
[ ] Southwest China
[ ] Northwest China

6.What is your ethnic group?

[ ] Han
[ ] Ethnic Minority: ____ (Please specify)

7.What is your average monthly income (in CNY)?

[ ] < 2,000
[ ] 2,000 - 5,000
[ ] 5,001 - 10,000
[ ] > 10,000

8.What is your type of medical insurance? (Select all that apply)

[ ] Basic Medical Insurance for Urban Employees
[ ] Basic Medical Insurance for Urban and Rural Residents
[ ] Commercial Health Insurance
[ ] No insurance (primarily self-pay)
[ ] Other Insurance: [Please specify in this area]
[ ] Prefer not to answer

9.How many internet-enabled devices do you regularly use?

*(e.g., Desktop PC, Laptop, Smartphone, Tablet, etc.)*
[ ] 1-2 devices
[ ] 3-4 devices
[ ] More than 4 devices

**Part Ⅱ Core Questions**

**Note**: Current applications of digital-intelligent healthcare include but are not limited to: Telemedicine, Remote Patient Monitoring, Robotic Surgical Assistants *(e.g., Dental Surgical Robots),* Rehabilitation Robots, Intelligent Diagnosis, Medical Image Classification for Lab Equipment & Scans, AI Patient Triage and Guidance Systems, Epidemic Surveillance, Portable/Wearable Devices *(e.g., for monitoring vital signs and personalized care)*, Public Hospital Internet Hospitals, Online Consultations *(e.g., using platforms like medGPT)*, Integration of AI with Traditional Chinese Medicine *(e.g., electronic pulse diagnosis)*, and AI Analysis of Laparoscopic Surgery Recordings, among others.

**Please read the following statements carefully and indicate your level of agreement by placing a checkmark (√) under the corresponding number.**

**1 = Strongly Agree**

**2 = Agree
3 = Neither Agree nor Disagree
4 = Disagree
5 = Strongly Disagree**

| **Statement** | **1** | **2** | **3** | **4** | **5** |
| --- | --- | --- | --- | --- | --- |
| 1. I have previously used digital-intelligent healthcare products or services in settings such as medical institutions or community health service centers, or during health education campaigns and disease screening programs. |  |  |  |  |  |
| 1. I perceive that digital-intelligent healthcare products or services would make my medical consultation process more convenient and the diagnostic outcomes more accurate. |  |  |  |  |  |
| 1. Using digital-intelligent healthcare products or services during medical consultations is convenient and does not require extensive technical knowledge. |  |  |  |  |  |
| 1. Learning how to use digital-intelligent healthcare products or services does not require a significant amount of time. |  |  |  |  |  |
| 1. If my family, friends, classmates, or colleagues were to use digital-intelligent healthcare products or services, I would be inclined to try them as well. |  |  |  |  |  |
| 1. Recommendations from healthcare professionals, such as doctors and nurses, would influence my decision to use digital-intelligent healthcare products or services. |  |  |  |  |  |
| 1. I would be highly likely to use digital-intelligent healthcare products or services if a family member had chronic health issues. |  |  |  |  |  |
| 1. In the future, I am willing to learn more about digital-intelligent healthcare products or services and their usage methods. |  |  |  |  |  |
| 1. With technological advancement, I am willing to accept the large-scale development of digital-intelligent healthcare in the future. |  |  |  |  |  |
| 1. I believe that an increasing number of people will choose digital-intelligent healthcare in the future. |  |  |  |  |  |
| 1. I enjoy using digital-intelligent healthcare products or services and maintain a positive attitude towards them. |  |  |  |  |  |
| 1. I am satisfied with digital-intelligent healthcare and its associated products or services. |  |  |  |  |  |
| 1. For the most part, my experiences with digital-intelligent healthcare have been pleasant. |  |  |  |  |  |
| 1. I am proficient in using digital-intelligent healthcare products or services. |  |  |  |  |  |
| 1. The available resources for digital-intelligent healthcare applications are abundant. |  |  |  |  |  |
| 1. I am sufficiently capable of overcoming various obstacles encountered while using digital-intelligent healthcare products or services. |  |  |  |  |  |
| 1. I am concerned about the potential security risks associated with using digital-intelligent healthcare products or services. |  |  |  |  |  |
| 1. Relatives, friends, and classmates around me are beginning to be exposed to, accept, or use digital-intelligent healthcare products or services. |  |  |  |  |  |
| 1. Public opinion and social trends influence my acceptance and use of digital-intelligent healthcare products or services. |  |  |  |  |  |
| 1. Relatives, friends, and classmates around me recommend that I use digital-intelligent healthcare products or services. |  |  |  |  |  |
