# Supplementary material for: Research on the implementation path of digital-intelligent healthcare based on the TAM model from the perspective of high-quality development
Source: BMC Health Serv Res. 2026 Mar 27;26:646. doi: 10.1186/s12913-026-14433-1 (PMC13151098; doi:10.1186/s12913-026-14433-1)
Supplement: Supplementary file 2 — Supplementary Material 2 [file 12913_2026_14433_MOESM2_ESM.docx]

Interviewee A: a student

**1. What is your major?**

I am a Health Management major.

**2. How many internet-enabled devices do you usually use?**

Four or five, including my phone, tablet, computer, and others.

**3. How is your physical health condition?**

Good.

**4. Have you recently been ill and visited a hospital? Do you go to the hospital frequently?**

Last year, I underwent a month of hospital treatment for a skin issue.

**5. During your medical visits, have you encountered any modern medical equipment?**

Yes, I have seen some instruments for examining the skin surface and epithelial tissues, such as skin CT and Wood's lamp.

**6. How familiar are you with these devices? (Have you seen or experienced them, or researched them online to learn more?)**

No, I have only seen them in real life and know what they are, but I haven’t looked them up online for deeper understanding.

**7. Have you heard of technologies like robotic surgical assistants, remote patient care, and monitoring?**

I've seen them online. For example, Apple's Vision Pro with its AR technology—I remember reading online about a hospital using AR during surgery to assist doctors in diagnosis.

**8. So you mainly learn about these through browsing the internet?**

Primarily through browsing, but I also get information from push notifications.

**9. Have you encountered these during your routine studies, such as in coursework or clinical hospital internships? (Coursework could refer to your current specialized subjects or extended courses that involve the application of such high-tech products.)**

I previously took an elective course related to artificial intelligence, which primarily covered topics like the Da Vinci Surgical System and AI-assisted diagnosis. I also learned about artificial hearts. For patients with heart conditions, they are indeed a great blessing, given that the number of heart transplants and donors is limited while the demand remains significant. Although artificial hearts may be relatively expensive, they at least offer a solution and a means to save lives—they represent cutting-edge, highly precise technology.

**10. Has anyone around you experienced such technologies?**

Not that I recall. Although these are high-tech products and their development is rapid, they are not yet widely accessible. Even some hospitals at the prefecture level lack such advanced equipment. Basic technologies like MRI, CT scans, and ultrasounds are more common, but cutting-edge technologies are rare. Additionally, the number of doctors proficient in using them is limited, and their coverage is not extensive. They are available in major cities like Beijing and Shanghai, but in remote areas such as Tibet and Xinjiang, they are seldom seen.

**11. Have you ever used online platforms for appointments or consultations?**

Nowadays, appointments are mostly made online rather than at the hospital. You can simply book via your mobile phone, which is more convenient and allows you to skip the queues.

**12. If you were to intern at a clinical hospital in the future, do you think the presence of high-tech equipment would be beneficial for your clinical internship?**

I am quite interested in new high-tech advancements myself, but as my major is not clinical, I would primarily focus on understanding what these technologies are rather than directly operating or using them. My involvement would likely be limited to learning theoretical knowledge and observing—more about seeing and experiencing rather than hands-on application.

**13. You mentioned previously having had a skin condition and experiencing treatment-related equipment. How would you assess its effectiveness compared to using only traditional medical methods?**

I believe high-tech diagnostic tools are more accurate. Nowadays, many doctors rely primarily on diagnostic equipment to assess conditions, as it’s difficult for the naked eye to determine the exact situation. For example, a skin CT can provide a clearer view of the deeper layers of the skin, helping doctors diagnose more effectively. Its role is primarily as an aid—ultimately, the doctor’s expertise is central—but such devices make the process more efficient and reliable.

**14. After observing doctors operate it, how would you describe the ease of use of the skin CT?**

It’s quite convenient, and the images are very clear.

**15. Were there any obstacles during its use?**

I have used a Wood's lamp, and there were basically no obstacles—it was very convenient. Its main function is to examine the skin. For example, vitiligo can be assisted in diagnosis with a Wood's lamp. The naked eye may not clearly see the extent of skin depigmentation, but the Wood's lamp can clearly show which areas of the skin have begun to turn white.

**16. What obstacles do you think we might encounter when experiencing or using smart devices in the future? (Whether you would find it difficult to accept, or you think others might)**

I don’t think I would have much difficulty accepting it, as I would actively try to learn how to use such smart devices. Nowadays, even blood pressure can be measured at home—I believe it’s worth learning. Having such devices at home helps you understand your own health condition to some extent. However, they can only serve as an aid and should not be relied upon entirely. The final diagnosis should still come from a doctor—we cannot completely depend on or trust smart products alone to diagnose our conditions. Therefore, combining them with a doctor’s experience is more effective. In the future, AI databases will likely surpass doctors in terms of knowledge—they will become more successful and mature. But at this stage, they remain primarily assistive tools. In terms of usability difficulties, the operational process—such as how to turn them on, what to do next after starting, etc.—should be simplified to better assist patients. For example, for health checks like measuring blood pressure, it should be as simple as wrapping the cuff around the arm and pressing a button to start. Since older adults may interact with these devices more frequently, making them simpler to operate would make them more welcome.

**17. Earlier you mentioned that if smart products become widespread in the future, you would actively try and embrace them. How do you feel about offline training or the use of such high-tech equipment in hospital outpatient settings?**

I hold a proactive, supportive, and approving attitude.

**18. What do you think the general public's attitude will be? What difficulties do you foresee in gaining public acceptance?**

I believe the public will gradually adapt to this trend. With the ongoing development of the era, it's a continuous process of advancement and renewal, so people will slowly come to accept and adopt these changes. However, at the current stage, it will likely be quite challenging due to societal aging—there is a significant elderly population that is still unfamiliar with using smart devices. Younger and middle-aged groups may find it easier, as smartphone usage is already widespread and they are accustomed to learning via their devices. Their learning curve is relatively lower. In contrast, for the elderly, both the learning cost and the willingness to adapt are likely to present greater difficulties.

**19. You mentioned supporting the promotion of such equipment in future medical education, hospital management, and clinical practice, as well as the inclusion of digital-intelligent healthcare knowledge in teaching materials. Based on your current understanding, what changes do you think the digital-intelligent healthcare system could bring?**

I believe it will make seeking medical care more convenient. If it can be fully popularized and promoted, it would be particularly beneficial for the elderly population. Not all elderly people have children to care for them—many live alone at home and may encounter various situations that require monitoring, supervision, or reminders. For example, there are already smart products that can remind them daily about which medications to take and in what dosage. However, these products are not yet widely adopted; only a few households have them, and older adults can only gradually learn and adapt to them. The majority of people still rely on hospitals, traditional treatments, and in-person appointments.

**20. If it becomes widespread in the future, would you be concerned about your privacy being compromised? (Assuming any illnesses you’ve had are recorded in the system and become publicly accessible information, would you mind or worry about that?)**

I certainly wouldn’t want others to know about any illnesses I’ve had. For example, when I previously had a skin condition, I didn’t want many people to find out, as others might mistakenly think it’s contagious or make assumptions about my health. Without understanding the condition, they might feel uneasy or even suspicious of me as a person. So I believe privacy protection is essential—it’s better to keep such information confidential. I hope the collected data can be properly managed and safeguarded, which largely depends on how seriously the companies designing these smart products prioritize and protect user privacy.
