# Supplementary material for: Research on the implementation path of digital-intelligent healthcare based on the TAM model from the perspective of high-quality development
Source: BMC Health Serv Res. 2026 Mar 27;26:646. doi: 10.1186/s12913-026-14433-1 (PMC13151098; doi:10.1186/s12913-026-14433-1)
Supplement: Supplementary file 3 — Supplementary Material 3 [file 12913_2026_14433_MOESM3_ESM.docx]

Interviewee B: a teacher

**1. Could you please briefly introduce the current academic research hotspots and trends in digital-intelligent healthcare within the nursing field?**

Over the past decade, the integration of technology and nursing has been steadily improving and becoming increasingly widespread, which is also closely related to national policies. In my recollection, around 2010, there were relatively few individuals engaged in the convergence of technology and nursing. Many believed that technological matters were the responsibility of IT professionals. However, in recent years, a growing number of nursing professionals have considered incorporating technology into nursing practice, engaging in both academic research and clinical innovation, which in fact can be seamlessly integrated. Currently, in terms of technological integration, the earliest adoption involved mobile information technology, which is widely recognized. Examples include smartphone-based applications, WeChat mini-programs, mobile networks, or mobile nursing carts equipped with PDAs used by clinical nurses. These were among the earliest technologies integrated into nursing practice and academia. Subsequently, advancements introduced virtual reality, wearable devices, big data analytics, and machine learning modeling for constructing predictive models. Up to the present, the integration of artificial intelligence has become more prominent. For instance, artificial intelligence encompasses various types, initially including robotics and intelligent decision support systems. In clinical settings, tasks such as patient diagnosis and risk assessment are now assisted by intelligent decision support systems.

Robotics is a familiar concept, and numerous robots related to healthcare and nursing are already in clinical use, with nursing professionals actively participating in their design and development. In recent years, particularly hot topics include generative artificial intelligence and AI digital humans. Generative AI gained widespread attention following the launch of ChatGPT in 2022, marking a significant milestone in the advancement of artificial intelligence. To date, mature examples of generative AI in China include Baidu’s ERNIE Bot, iFLYTEK’s Spark, and Tsinghua’s Zhipu Qingyan, among others. With the development and maturation of these domestic AI technologies, they are increasingly being integrated into nursing for innovative applications. AI digital humans have also become a major focus in recent years. They can be utilized for science popularization, training, educational reform, as well as digital twin applications, patient monitoring, early warning systems, remote management, and more. These technologies are rapidly evolving. The integration of such technologies into nursing is becoming an increasingly prevalent trend. Last year’s National Two Sessions emphasized the construction of a digital China, and this year’s sessions highlighted the development of new quality productive forces, both underscoring the integration of technology across various industries. From a nursing perspective, we actively embrace the guidance of the times and policies to advance the field of digital-intelligent healthcare, which aligns with international trends. Increasingly, technology is being integrated and innovated within nursing across broader scopes.

**2. The application of digital-intelligent healthcare may need to be further promoted in clinical and pre-hospital settings. What challenges do you think the training of nursing and medical students faces under such trends, particularly in areas such as curriculum design and practical training?**

Since the School of Nursing at Capital Medical University pioneered the integration of technology and nursing in China—starting our efforts in this area as early as 2010—we have been paying close attention to this issue from a very early stage. Our related academic fields are also at the forefront nationally. In this regard, we have established a Nursing Informatics course at the undergraduate level and integrated digital-intelligent nursing courses at the graduate level, all aimed at cultivating the mindset and innovative capabilities for integrating digital-intelligent technology with nursing among both undergraduate and graduate students. Therefore, we are relatively ahead in this field.

From the perspective of medical students' development, they face significant challenges but also great opportunities. I believe young people are inherently digital natives, with a rapid ability to embrace and master technology. As long as they are willing to enhance their skills in integrating technology, they can certainly achieve this and excel at it. We deeply understand that to effectively integrate technology with healthcare and nursing, especially in keeping pace with the changes of this era—whether actively or passively—we must adapt to the characteristics of the times. In this context, rather than being passive, we should strive to be active participants, designers, and leaders.

I believe that our students at Capital Medical University, on such a platform, with our nursing discipline being nationally recognized as a top-tier discipline and given our national standing and standards, should also be at the forefront, both domestically and internationally. To excel as healthcare professionals in the digital age, one must first maintain sensitivity and curiosity toward technology. This is crucial because you must be interested and curious about technological advancements to proactively understand them and find ways to integrate them into healthcare practice. Thus, I believe sensitivity and curiosity are foundational.

Secondly, having a strong innovative mindset and awareness is essential. Once we understand these technologies, we must constantly think about how to integrate them into healthcare and nursing to serve our needs, innovate our practices, and generate academic outcomes. Thirdly, I believe it is about the real execution capability in digital-intelligent healthcare and nursing—meaning not only having the mindset and awareness but also sufficient action. In academic research in this field, innovative ideas are vital; having an innovative idea is already half the success. But what does the other half depend on? It depends on execution. Many people in the world are innovating. We might think of a clever integration of a technology with a specific health issue, but others might have the same idea. What matters then is execution and speed. Even if we have the idea, if we act slowly and fail to implement it promptly, we may be surpassed by others.

From the perspective of the entire medical field, no matter who takes the lead, it represents overall success for the medical community. However, on an individual level, whether in the integrated development and innovation of digital-intelligent healthcare and nursing or in other work and studies, the same principle applies: execution is crucial. Being able to implement ideas quickly, accurately, and rigorously, and promptly achieve innovative results and outcomes, is essential. This is also characteristic of our era, which moves at a very fast pace. Everything emphasizes effectiveness—not just having ideas but taking action, rather than remaining stagnant for a long time without implementation, which yields no results.

Therefore, I believe these points are crucial: sensitivity and curiosity, maintaining an innovative mindset and awareness, and having strong execution capabilities. From a methodological perspective, we must also possess innovative abilities and methods. As young people and digital natives, I believe you are not lacking in methods or techniques. The internet offers a wealth of free learning resources, and as long as you want to learn, you can find the resources and methods. The question is whether you are willing, able, and eager to learn. So, if I were to add a fourth point, it would be innovative abilities and skills. This involves methodological aspects, but these can be acquired and improved through learning and practice.

**3. As you mentioned, our nursing program is at the forefront nationally in integrating digital-intelligent healthcare. Could you provide some examples of how we have incorporated digital-intelligent healthcare into our nursing curriculum?**

Let me give a few examples. First, let’s talk about virtual reality. When you think of virtual reality, what comes to mind? Is it wearing VR glasses and immersing yourself in a virtual world? Actually, virtual reality comes in many forms. Initially, there was desktop virtual reality, where you used a mouse, stylus, or touchscreen to interact with a virtual system on a screen while remaining in the real world—this was the earliest application of VR. Nowadays, immersive virtual reality is widely used across many industries, including nursing and healthcare. It involves putting on VR glasses to enter a virtual space, which could be an operating room, an emergency scenario, a hospital ward, or a community setting. Essentially, you enter a completely virtual world that is largely disconnected from the real world, allowing you to learn relevant skills and techniques. This is the most common application.

However, the teaching methods developed by our team go beyond such virtual systems for training students’ comprehensive skills. We also use mixed reality technology, which is a branch of virtual reality that has evolved to a more advanced stage. So, what is mixed reality? For instance, much of nursing training involves using simulated models, such as practicing CPR on a mannequin. Even if the mannequin has highly realistic skin and accurately mimics human anatomy, it is still a simulation. Take CPR as an example: during training, we practice chest compressions on a mannequin, pressing down with adequate frequency and depth. Advanced mannequins may have automatic timers that count compressions and indicate appropriate depth, but that’s about it. What we do with mixed reality is different. For example, since our team focuses on maternal and child health, we have developed several mixed reality systems. In CPR training, when you press down on the mannequin, you only see the chest wall moving up and down, with no visibility into how the heart changes internally—even in real-life CPR training, we cannot see the heart due to the skin and chest wall. With mixed reality, students wear MR glasses while standing next to the mannequin. During practice, they see the mannequin’s chest wall, but through the virtual glasses, they can also visualize a heart, blood vessels, and blood flow beneath the chest wall. They can observe whether the heart is beating and how it responds to compressions—seeing the heart compress and blood flow with each press. This seamlessly integrates abstract physiological processes and structures with the physical mannequin, allowing students to observe all physiological changes during practice.

In obstetrics, for instance, when a fetus is in the mother’s womb, how do doctors examine it? There are specific palpation techniques to determine whether the fetus is head-down or breech, locate the buttocks, back, and limbs—all without visual access, relying solely on skilled touch. Traditionally, students practiced on simulated models to learn these steps. Now, with MR glasses, students palpate the model’s abdomen while seeing a virtual representation of the fetus inside. How is this achieved? It’s about perfectly merging virtual simulations with physical models, enabling students to simultaneously feel the tactile feedback of the model and visualize the virtual organs. We’ve developed many mixed reality teaching systems to help students practice skills and steps while understanding how each action affects real physiological functions. This integration makes it much easier for students to grasp the relationship between abstract knowledge and practical skills.

Then there’s AR technology, or augmented reality. Whether using MR or traditional VR, these are often confined to laboratory settings. However, augmented reality has unique features, such as the ability to embed it in QR codes. During lectures or in any setting, students can scan the code to access virtual learning content on their phones, enabling flexible, anytime-anywhere virtual learning. This is far more adaptable than requiring specific spaces or equipment.

Therefore, our use of virtual reality technology, particularly the latest innovations, drives teaching reforms and talent development. Students not only acquire professional knowledge but also learn through technology, which greatly enhances their technological sensitivity and hands-on experience. Many students, guided by faculty, have created numerous inventions. How did they come up with these ideas? By engaging with technology during their studies, they began exploring how to integrate it with other clinical issues to develop new innovations. This creates a mutually beneficial cycle of teaching and learning. While our primary goal is professional nursing education, we are also equipping students with technological literacy.

**4.In the context of pre-hospital care, what is the acceptance level of digital-intelligent healthcare among the elderly?**

This question relates to the target population of nursing services. Whether in clinical or community settings, our service recipients are the general public, including both patients and healthy individuals under community care. When categorizing this population by age, young and middle-aged individuals generally exhibit better technological awareness and higher acceptance. However, the elderly do face challenges in adopting digital-intelligent healthcare. Considering that we are in the era of 'Digital China,' where the nation advocates for 'enhancing digital literacy for all,' adapting to this trend—whether actively or passively—is essential.

Similarly, for the elderly, on one hand, proactive efforts are needed to teach them basic digital-intelligent skills, such as using smartphones for medical appointments or accessing test reports. Many healthcare services they receive are digitally delivered through technology integration, making it necessary for them to possess certain digital capabilities. This is not solely the responsibility of healthcare professionals. Building an 'age-friendly' society requires shared responsibility from families and society as a whole. For instance, adult children have a duty to teach their elderly family members basic digital survival skills, as technology integration extends beyond healthcare to daily activities like dining and shopping. At the same time, the elderly should also take the initiative to learn these skills.

From the perspective of healthcare providers, when developing products or technologies for the elderly, it is essential to prioritize user-friendliness through 'age-appropriate design.' This means creating systems that are as intuitive as possible—for example, using larger fonts to accommodate the visual needs of the elderly and simplifying system structures to avoid excessive nested modules. System design should consider the distinct needs of children, youth, middle-aged individuals, and the elderly. For children, interactivity and gamification are key; for the elderly, clarity and simplicity are paramount.

In summary, addressing this issue requires a two-way effort: the elderly must enhance their digital-intelligent literacy, while all tools, products, and technologies should be designed to be straightforward and easy to use. This is a mutually reinforcing process.

**5. Regarding the challenges faced by nursing staff in enabling the elderly to develop digital literacy, what are the main difficulties, and is there resistance from some elderly individuals toward digital-intelligent healthcare?**

Currently, many of our services and research initiatives primarily target elderly users. During the promotion of these services, we do encounter difficulties in terms of their acceptance and usage. The country is currently undergoing a transition from partial to universal digital-intelligent adoption, and while some elderly individuals are quite adept and possess basic digital literacy when introduced to digital-intelligent products, others still use basic non-smartphones, commonly referred to as ‘senior phones.’

Over the years, some elderly individuals in suburban areas may still rely on such basic phones, but the majority now use smartphones and are capable of using applications like WeChat—this reflects the natural progression of the times. Beyond the issue of unfamiliarity with technology among the elderly, hardware accessibility also poses a challenge. The former relates to a lack of foundational digital skills, while the latter involves difficulties in downloading and using applications, especially since we cannot provide every elderly person with a smartphone.

In previous related studies, we implemented a strategy of distributing smartphones uniformly to elderly participants and collecting them after the study concluded. The findings revealed that, with proper guidance, these elderly individuals were able to overcome some of the challenges associated with smartphone use. Prior to this, their lack of digital literacy was largely due to economic constraints limiting their access to such devices.

In recent years, as smartphones have become more affordable and widespread, hardware-related issues have gradually diminished, revealing instead a greater need to address the digital literacy and capabilities of the elderly. In both research and clinical practice, we encounter challenges related to the use of smart devices by older adults. To tackle this, we assign dedicated personnel to teach them and adapt devices to be more senior-friendly. Given the limited sample size of elderly participants in studies, personalized instruction is often employed. Due to the close connection between nursing and clinical practice, our teaching is guided by clear research objectives and supplemented with specially designed manuals for elderly users.

If elderly individuals forget certain operational details after instruction, we utilize voice-assisted technology to provide reminders. For instance, by tapping embedded audio icons or scanning QR codes in the manuals, they can listen to standardized, easy-to-understand explanations recorded by nurses. Beyond manuals, voice-assisted applications also include instructional videos, posters, and memos. The content covers medication instructions, healthy eating habits, exercise methods, and more. Additionally, WeChat mini-programs are available for elderly users to seek online consultations after returning home.

With the goal of making services more accessible, we teach older adults some basic skills that are simple to apply without requiring significant effort. However, it's important to note that this is not a nationwide initiative—our services are limited to specific groups. For example, if I work in a stroke ward, our services are confined to the elderly stroke patients in those wards. Achieving widespread digital literacy among all elderly individuals falls more under the scope of a 'national campaign,' while healthcare professionals focus on more targeted populations.

When collaborating with urban hospitals, the service recipients typically come from nearby urban areas, where their knowledge and cultural literacy are relatively higher, making instruction easier. In contrast, in some suburban hospitals, patients often come from surrounding rural areas with lower educational backgrounds. They tend to delay treatment until their conditions worsen, showing less proactive engagement with their health and lower motivation to learn. Such groups are generally less inclined to adopt digital healthcare technologies and learn at a slower pace.

In summary, healthcare professionals primarily operate within medical institutions, serving well-defined user groups whom they can effectively teach. Achieving universal digital literacy among the broader population is challenging due to regional, generational, and institutional disparities. Considering the mobility of patients, healthcare workers are also making significant contributions to the development of a 'Digital China.' If nationwide digital literacy initiatives can elevate the overall digital competence of the public, it would undoubtedly bring great convenience to the healthcare and daily lives of the elderly. As digital literacy improves and becomes more widespread, the time and effort required for teaching will gradually decrease.
