# Supplementary material for: Research on the implementation path of digital-intelligent healthcare based on the TAM model from the perspective of high-quality development
Source: BMC Health Serv Res. 2026 Mar 27;26:646. doi: 10.1186/s12913-026-14433-1 (PMC13151098; doi:10.1186/s12913-026-14433-1)
Supplement: Supplementary file 4 — Supplementary Material 4 [file 12913_2026_14433_MOESM4_ESM.docx]

Interviewee C: a student

**1. I understand you're currently a member of your school's track and field team. How would you assess your current health status?**

My health is generally good. Thanks to regular running, I maintain a relatively lean physique.

**2. How often did you visit the hospital in the past?**

I used to go to the hospital relatively frequently because I have some minor stomach issues.

**3. Are you familiar with the modernized appointment registration process nowadays?**

In the past, registering for appointments often required standing in long queues, which always made hospital visits feel inconvenient. However, with the introduction of online appointment registration later on, the process has become much more convenient.

**4. You mentioned examples like printing medical reports using machines, which indeed fall under digital-intelligent healthcare. Beyond that, we often discuss technologies such as robotic surgical assistants, remote nursing monitoring, AI analysis of laparoscopic surgery recordings, and even AI-assisted pulse diagnosis in traditional Chinese medicine. Have you heard of such digital-intelligent healthcare services?**

Yes, I have. For instance, at a school seminar I attended this afternoon, a paramedic shared her experience: she once received an emergency call about an elderly person who had no heartbeat. Through telemedicine, she guided the family to perform CPR, and the patient regained consciousness within a minute.

**5. Based on your experience, how would you describe your understanding and impression of devices or technologies representing digital-intelligent healthcare?**

In my understanding, digital-intelligent healthcare primarily involves the integration of big data and artificial intelligence. For example, as someone with a stomach condition requiring medication, I used to forget to take my pills regularly. Later, I learned that smart bracelets can send medication reminders, which has since helped me adhere to my schedule much more consistently.

**6. Have any classmates or family members recommended digital-intelligent healthcare-related content to you?**

My family members are relatively less familiar with this field. However, one of my classmates, who is very interested in healthcare, has conducted research on robotic surgery and shared insights with me.

**7. Have you personally engaged in any practical activities or research related to this field? Or have you observed how people around you use such devices or technologies?**

One of my relatives, who is an elderly person living alone and has diabetes, relies on a mobile app to receive medication reminders from her children. The app also allows for real-time monitoring of her blood sugar levels.

**8. Apart from smart bracelets, have you experienced any digital-intelligent healthcare services in hospitals?**

Recently, when I went to the hospital for an eye exam to update my glasses prescription, the doctor first used a machine to take a preliminary measurement of my vision, followed by a more precise manual adjustment. This process made the examination faster and more accurate.

**9. Are you familiar with hospital management models or related aspects?**

Not really.

**10. Even without in-depth knowledge, can you imagine how digital-intelligent healthcare could be applied to hospital management or patient care processes?**

I think medical records of many individuals could be uploaded to a big data platform. By analyzing correlations within the data, a standardized public healthcare protocol could be developed. If everyone follows such a protocol, it might help improve the effectiveness of public health initiatives in the future.

**11. As a medical student, what kind of teaching methods do you prefer in your classes?**

I prefer having instructors guide us through theoretical knowledge first. Once we have a solid grasp of the theory, we can then proceed to laboratories or specimen rooms for hands-on experiments and structural observations.

**12. If digital-intelligent healthcare technologies were integrated into daily classroom learning, do you think it would impact or assist your studies?**

I believe it would have a significant impact. Since we cannot directly observe internal structures of the human body, technologies like the 3D modeling machines in our school—which allow us to view detailed and precise anatomical structures—would help me develop a more comprehensive understanding.

**13. How widespread do you think digital-intelligent healthcare is among people around you? How familiar are they with this concept, and has anyone actively discussed it with you?**

Awareness is somewhat limited. Apart from the classmate I mentioned earlier, not many people are familiar with it or engage in discussions about it.

**14. In your current use of digital-intelligent healthcare devices, such as the smart bracelet and blood glucose monitoring you mentioned, have you encountered any obstacles?**

Yes. I feel that the current devices are not yet precise enough and cannot provide continuous, real-time measurements.

**15. What is your overall attitude and expectation toward the development of digital-intelligent healthcare?**

I hope it can become more advanced, refined, and personalized. For instance, procedures like gastroscopy can be quite uncomfortable now. I wish there could be a very tiny probe for monitoring, so people wouldn’t have to endure the discomfort during such examinations.

**16. How do you feel about the widespread adoption of digital-intelligent healthcare technology in medical schools?**

I hope it can help more people gain a better understanding of this field.

**17. What is the fundamental reason behind your consistently positive attitude toward digital-intelligent healthcare?**

I simply hope it can benefit more people, as there are indeed many facing health issues right now.

**18. With the development of these new technologies, would you be willing to recommend digital-intelligent healthcare to friends and family around you?**

I’m quite willing to do so. Once I have a deeper understanding of this area, I’d be happy to introduce and recommend it to others.

**19. Do you have concerns about the security of digital-intelligent healthcare, such as privacy issues?**

Yes, I do. For example, whether medical data uploaded to systems might be leaked.

**20. Do these concerns affect your previously positive attitude toward it? Do you have more worries or more support?**

I still support it more. I believe that as technology advances, confidentiality will improve, and there will also be relevant laws and regulations in place.

**21. Apart from privacy issues, do you have any other concerns about digital-intelligent healthcare? For instance, it might be difficult to control, or elderly people may struggle to use it, etc.**

I think there are indeed challenges for the elderly, as some may not be familiar with using smartphones or other smart devices. This is also true for elderly people with conditions like Alzheimer’s disease. However, as technology evolves, solutions may emerge—for example, their children could remotely assist them without requiring the elderly to operate the devices themselves.
