# Supplementary material for: Research on the implementation path of digital-intelligent healthcare based on the TAM model from the perspective of high-quality development
Source: BMC Health Serv Res. 2026 Mar 27;26:646. doi: 10.1186/s12913-026-14433-1 (PMC13151098; doi:10.1186/s12913-026-14433-1)
Supplement: Supplementary file 5 — Supplementary Material 5 [file 12913_2026_14433_MOESM5_ESM.docx]

Interviewee D: a teacher

**1. Could you share the current hotspots and trends in the field of digital-intelligent healthcare, particularly concerning medical colleges and universities?**

It may involve several aspects. First and foremost, digital technology applied in the healthcare field is actually a process. In its earliest stages, it was referred to as digital technology, which later evolved into digital-intelligent technology. Here, 'digital-intelligent' combines digital and intelligent elements, with the 'intelligent' aspect specifically incorporating artificial intelligence. Prior to this, digital technology primarily encompassed the application of foundational technologies such as cloud computing, big data, the Internet of Things, and big data analytics—this was the digital era. Now, in the digital-intelligent era, it essentially integrates artificial intelligence, machine learning, planning algorithms, and currently popular large-scale models, all applied within the healthcare sector. Therefore, if we are to identify the most significant hotspots in recent years, they undoubtedly revolve around the application of technologies like large-scale models, generative artificial intelligence, and similar machine learning advancements in healthcare, with a remarkably broad scope of application.

If your focus is on healthcare, I believe the application of digital-intelligent technology in this field can be categorized into three main functional scenarios:

First, it can enhance quality—improving the quality of healthcare services. This includes technologies that assist physicians in diagnosis, as well as current applications of large-scale models in new drug research and development. It also involves generating personalized health profiles for health management, all falling under the umbrella of quality improvement.

Second, it can increase efficiency—enhancing effectiveness. For example, physicians are often extremely busy, and their time is highly valuable. Now, technologies can automatically generate electronic medical records. For primary-level hospitals, AI can enable intelligent follow-up, such as making phone calls to patients to provide health guidance or consult on certain issues. These tasks, which would typically be handled by doctors or nurses, can now be assisted by artificial intelligence, thereby improving work efficiency and reducing the burden on medical staff. This constitutes the second category.

Third, it can address the issue of uneven distribution of medical resources. This includes telemedicine, internet-based diagnostics and treatment, and intelligent diagnostic assistance technologies for primary-level doctors. All of these can help institutions or medical personnel with limited capabilities or limited access to high-quality medical resources enhance their service capacity. Training primary-level medical personnel takes time, but with technological support, they can quickly achieve a certain level of service quality.

Thus, the application of digital-intelligent healthcare essentially revolves around these three dimensions. At its current stage of development, artificial intelligence also requires support from other technologies. For instance, collecting vast amounts of data—such as health monitoring data—relies on Internet of Things technology. Once collected, where is this data stored? This necessitates cloud computing to enable mobile storage. To utilize this data effectively, big data methodologies are required for analysis. Through the process of big data analysis, algorithms are gradually refined, giving rise to machine learning algorithms.

Additionally, in other applications within healthcare, technologies such as robots or intelligent robotic arms may emerge to assist doctors in achieving more human-like applications.

I believe the current research hotspots and application scenarios are largely centered around these areas.

**2. When interviewing doctors, some have mentioned that using tools like electronic medical records feels less convenient in certain aspects compared to the previous handwritten methods. Additionally, they noted that some digital-intelligent healthcare operations still require further learning. What are your thoughts on how we should train medical students to address such situations?**

Medical students do require training in digital-intelligent technologies to enhance their digital literacy, or so-called AI literacy. On one hand, as students, they currently rely on various tools for learning. Improving learning efficiency and research productivity often depends on the support of digital technologies. For example, when writing literature reviews nowadays, it may no longer involve manually reviewing each paper one by one. Instead, tools can be used to generate intelligent summaries or abstracts of literature, thereby enhancing their management capabilities and efficiency. Similarly, when writing or revising papers, generative artificial intelligence can provide significant assistance, such as making students’ English expression more fluent with minimal effort. This also includes programming skills, where AI can help them complete their studies and research more effectively.

On the other hand, once they become doctors, large hospitals are rapidly advancing in terms of informatization. Internationally advanced information systems or technological applications are quickly being implemented in hospitals, but doctors may not necessarily be able to accept or effectively utilize them. In this context, if one can quickly adapt to these technologies, it can significantly improve work efficiency.

Moreover, even after becoming doctors, they may engage in research, such as analyzing gene sequences or conducting intelligent diagnostics. These tasks require a perspective rooted in digital-intelligent technologies. They need to understand which cutting-edge technologies can help improve the quality or accuracy of surgeries in their specific field—for example, a gastroenterologist should be aware of models applicable to their area. They should possess this kind of sensitivity. Therefore, I believe medical students indeed need such training to establish a foundational literacy in this regard.

**3. As previously mentioned, digital-intelligent healthcare indeed contributes to enhancing diagnostic and treatment efficiency, as well as learning productivity. However, an interesting phenomenon observed in recent years, echoed by many doctors we interviewed, is that the pervasive nature of internet-connected tools has blurred the boundaries between work and personal life. For instance, due to the elimination of geographical constraints, tasks that once had to be completed at the hospital or workstation can now be addressed from home. This unintentionally increases their workload, as they frequently receive work-related messages even during off-hours. Additionally, concerns have been raised about data security risks and the reliability of remote diagnostics. What are your thoughts on the safety and security measures for artificial intelligence in this context**?

The development of any new technology follows a certain process. Initially, it inevitably has shortcomings or imperfections. However, with rapid technological advancement, continuous efforts are made to address and improve these issues. For example, while AI in healthcare may currently face challenges such as lower diagnostic accuracy, training large models with more domain-specific medical data can enhance their intelligence. If AI’s diagnostic accuracy eventually surpasses that of human doctors, it could become a valuable tool to leverage.

Regarding the concerns you mentioned—such as doctors feeling overwhelmed by the blurring of work-life boundaries—it's natural to have such anxieties. The pace of work is fast, and pressure is high. However, technological progress is a double-edged sword: on one hand, it improves efficiency; on the other, it may intensify workloads and expectations. Still, attitudes vary. Those who embrace innovation may welcome these changes, while more traditional individuals might prefer familiar methods, like handwritten records. But in today’s world, insisting on handwriting is hardly feasible—imagine doctors in clinics handwriting records while patients queue endlessly. Ultimately, the benefits outweigh the drawbacks. The key is to address challenges proactively, refine the technology, and enhance overall service quality and efficiency, while also considering the practical realities for medical professionals.

**Interviewer:** As you mentioned, by strengthening digital literacy training for medical students, their mindset may gradually shift, making them more receptive to digital-intelligent healthcare.

**Interviewee:** Exactly. It’s like how we discuss aging populations today—older adults often struggle to adapt to new technologies, finding them difficult to learn or use. But as generations shift, those who grow up with these technologies will naturally embrace them. Technological evolution progresses generation by generation. Your generation, for instance, is learning AI-related skills, which are more advanced and complex than the internet technologies we adapted to. When you grow older, you’ll likely find AI or robotics perfectly acceptable, whereas many in my generation might still feel uneasy about robots providing care or assistance. This is simply how human society moves forward—from the steam era to the electrical era, and now to the information age.

I often share an example with my students: the first general manager of IBM once said, when the world’s first personal computer was produced, that “five computers worldwide would be enough.” Today, that statement seems laughable. But in his time, that was the limit of his vision—he couldn’t imagine everyone needing such computing power, believing only governments or armies might require it. Yet look at us now: personal computers are everywhere, and individuals own multiple devices. Technology evolves generation by generation, constantly reshaping what we consider possible.

**4. You mentioned the issue of acceptance among older adults earlier. In hospitals, even small devices like self-service check-in kiosks can be challenging for them to use independently, often requiring assistance from family members. Given that older adults currently make up a significant portion of hospital visits, do you think it is more appropriate to promote digital-intelligent healthcare for this group or to continue retaining traditional offline registration methods?**

Both approaches are indeed necessary. Offline services must certainly be retained—as mentioned earlier, people vary significantly. Some elderly individuals are perfectly capable of learning new technologies. I recall reading about how a person has both a chronological age and a psychological age, and the latter is closely linked to how frequently they update their electronic devices. If someone regularly adopts new gadgets, their psychological age tends to be younger, even if they are in their sixties or seventies. So, even among the elderly, those who are willing to learn can certainly do so. However, many older adults either find it difficult to adapt or prefer not to engage, often due to more conservative mindsets. Therefore, it is essential to maintain alternative pathways. Even if only a few people rely on them, we should still provide dedicated registration counters or human-assisted service windows for the elderly."

**Interviewer:** So, it's not only about individualized approaches but also about incorporating humanistic care into the pre-hospital patient journey.

**Respondent:** Exactly, yes. This is fundamentally an issue of equity and inclusivity.

**5. Returning to the topic of medical student education, we surveyed some medical students from our institution. Most indicated that their peers and family members have little understanding of digital-intelligent healthcare and rarely discuss it with them. While they generally hold a positive attitude toward digital-intelligent healthcare themselves, their perceptions remain vague. In your view, how should we advance the education on digital-intelligent healthcare for medical students in the future?**

Our country is already working on this, isn't it? For instance, our university is about to introduce a general course on artificial intelligence for all students across all majors. That’s how we’re rolling it out step by step.

**Interviewer:** “So, should we aim to integrate digital-intelligent healthcare into all courses at once, or adopt a more gradual approach?”

**Interviewee:** “It should definitely be gradual. After all, this technology—no matter how advanced—isn’t capable of doing everything. It remains primarily an assistive tool.”

Interviewer: “So it’s still seen as a tool, not as a standalone subject to be studied in depth. The focus remains on its integration with medicine, with medical expertise itself at the core.”

**Interviewee:** “Exactly. Moreover, we’re also operating within a broader global context. The country is keenly aware of competitive pressures—if we fall behind in this wave of AI-driven technology, we could lag significantly. As we discussed earlier, from the steam era to the electrical era, then the information era, and now the intelligent era—each wave of technological revolution reshapes economies, societies, and even the global landscape. The state recognizes this potential risk, which is why it emphasizes that the new generation of students must learn about and understand these technologies. Only then can we ensure we don’t fall behind.”
