# Supplementary material for: Research on the implementation path of digital-intelligent healthcare based on the TAM model from the perspective of high-quality development
Source: BMC Health Serv Res. 2026 Mar 27;26:646. doi: 10.1186/s12913-026-14433-1 (PMC13151098; doi:10.1186/s12913-026-14433-1)
Supplement: Supplementary file 6 — Supplementary Material 6 [file 12913_2026_14433_MOESM6_ESM.docx]

Interviewee E: a doctor

**1. Have you ever had any understanding of “digital-intelligent healthcare”?**

Since we are in the endocrinology department, our primary focus is on diabetes. As a chronic disease, diabetes particularly requires long-term management of patients. For such long-term management, it is difficult for patients to frequently visit the hospital. Therefore, we often rely on remote technologies to manage patients, such as chronic disease management systems, diabetes management systems, and post-discharge regular follow-ups. In essence, the nature of these technologies is quite similar.

**2. Could you provide some examples of how these technologies have enhanced medical efficiency or patient experience in practical applications?**

For instance, our chronic disease management system has, to some extent, reduced the workload for doctors and improved the healthcare experience for patients. Since patients are not required to visit the outpatient clinic in person, they can instead raise questions to their responsible doctors through the system based on their individual conditions. For doctors, the system allows us to preview all of a patient's medical records, test results, and examination reports from the hospital, thereby enhancing our understanding of the patient's condition. Thus, it significantly enhances convenience in clinical practice.

**3. Have you encountered any challenges while utilizing these digital-intelligent healthcare technologies?**

For elderly patients, the operation can be relatively cumbersome. This is particularly evident in grassroots healthcare settings, where diabetes patients are predominantly middle-aged and elderly individuals who often have relatively lower levels of digital literacy. Under such circumstances, these technologies are not very user-friendly for older patients. For physicians, however, this issue is less significant. Although there are both older physicians and younger physicians, since computers have long been integrated into our daily work, challenges in this regard are not substantial.

**4. What are your suggestions regarding data security?**

We haven't given much thought to data security, as we operate in such a big data environment where maintaining privacy is quite challenging.

**5. How would you evaluate the simplicity of operation and user-friendliness of the current digital-intelligent healthcare system? Do you find it sufficiently convenient?**

It is definitely not sufficient, though this involves many detailed aspects of design and implementation. At first glance, the overall system may seem acceptable, but in reality, it still lacks refinement in many details. Additionally, since our hospital has implemented such a system, we doctors are required to proactively provide online care and support to patients. This involves using our spare time to respond to inquiries and manage cases, which has, to some extent, increased our workload compared to before.

**6. You mentioned earlier that some patients are unfamiliar with digital-intelligent healthcare. Does this emerging technology affect communication and the relationship between you and your patients?**

No, it doesn't. We respect the patients' preferences. In this regard, patients also have a certain degree of autonomy. If a patient is unwilling to use such technology, we will not insist.

**7. What suggestions do you have for improving the functionality of digital-intelligent healthcare?**

We hope it can be made simpler and more powerful in terms of features. For example, it could allow for patient categorization, enabling doctors to classify patients based on different conditions such as the type of diabetes, age, and complications. Additionally, we hope these systems can provide more technical support for doctors. For instance, managing diabetic patients requires extensive knowledge about medication adjustments. It would be helpful if the system could supply such information to patients directly, eliminating the need for doctors to type it out or repeatedly explain it. Overall, we still hope it can be made more user-friendly.

**8. The country is currently advocating for the "high-quality development" of healthcare and has introduced many policies. What suggestions do you have regarding the current policy environment for the development of digital-intelligent healthcare?**

In the current context of rapid informatization, the state has provided substantial support and strong advocacy. However, at the grassroots level, top-down implementation and promotion still require time. Additionally, in terms of system optimization, less developed regions tend to adopt such technologies later compared to major cities. Moreover, we often hope for an "integrated healthcare network" that would allow us, even in outpatient settings, to access the hospital's final decisions on patients and the integration of all their medical information.

**9. As digital-intelligent healthcare technology continues to evolve, what are your thoughts or expectations for emerging technologies?**

For now, I have no additional expectations beyond hoping to provide more convenience for both patients and healthcare professionals. For example, our current system, in my opinion, has become more cumbersome compared to the previous handwritten approach. While these systems have been introduced, they have correspondingly introduced certain challenges.

**10. In this context, do you think the acceptance and willingness of clinical doctors to use digital-intelligent healthcare will be affected?**

This will not be the case, as its benefits certainly outweigh the drawbacks.

**11. What suggestions do you have regarding training and education in digital intelligence for clinical doctors?**

I don’t have many specific suggestions in this regard. Whenever a new system is launched, we provide multiple training sessions for our staff and users.
