# Supplementary material for: Research on the implementation path of digital-intelligent healthcare based on the TAM model from the perspective of high-quality development
Source: BMC Health Serv Res. 2026 Mar 27;26:646. doi: 10.1186/s12913-026-14433-1 (PMC13151098; doi:10.1186/s12913-026-14433-1)
Supplement: Supplementary file 7 — Supplementary Material 7 [file 12913_2026_14433_MOESM7_ESM.docx]

Interviewee F: a doctor

**1. Does your hospital currently offer an integrated online-offline registration system?**

Yes.

**2. In your daily observations, are these registration machines and processes convenient for elderly patients?**

Registration for elderly patients is often handled by their family members.

**3. So, would you say that elderly patients generally lack sufficient digital literacy?**

Most do.

**4. How widely are electronic medical records, telemedicine, and similar methods utilized in your department?**

Our hospital has adopted internet-based healthcare services. Attending physicians use them to communicate with patients, including for remote consultations.

**5. Could you provide some specific examples of these applications?**

For instance, just yesterday our hospital conducted an internet-based consultation with Wuwei People’s Hospital in Gansu Province to assist them in reaching clearer diagnoses.

**6. In your current clinical practice, have you encountered any difficulties in promoting digital-intelligent healthcare? For instance, issues like data security, or acceptance from doctors and patients, etc.**

In reality, I believe patients are generally receptive, as digital platforms can help them receive diagnosis and treatment more conveniently and efficiently. For doctors, it works well for simple conditions, but for complex diseases, accurate diagnosis online remains challenging, and in-person consultations are still more reliable.

**7. For elderly individuals, often referred to as “digital immigrants” in this era, you mentioned they usually rely on family members for registration. How would you describe their acceptance of digital-intelligent healthcare?**

They tend to feel rather confused or uncertain about it.

**8. In your work, do you feel that digital-intelligent healthcare improves your efficiency or aids in diagnosis?**

Since it primarily serves the patients, I haven’t felt a significant increase in my own efficiency. However, from the patients' perspective, it certainly offers greater convenience.

**9. This might lead to the topic of medical training. Do you think it’s necessary to organize centralized learning sessions for doctors on digital-intelligent healthcare?**

Yes. Tools like Tencent Meetings are already forms of digital-intelligent healthcare, and even offline medical training now frequently incorporates such applications.

**10. Are you suggesting that digital-intelligent healthcare might be more readily accepted by patients, while doctors may find it relatively more challenging?**

Not exactly “challenging”. Digital-intelligent healthcare makes consultation times more flexible—for example, some meetings can be scheduled in the evening, which is convenient. However, it also means doctors may need to invest more time, blurring the boundaries between work and personal life.

**11. So, these challenges stem from the inherent nature of the internet itself rather than technical issues with digital-intelligent healthcare operations, is that correct?**

This aspect also requires improvement. For instance, during remote consultations, patient medical records aren't always directly accessible—sometimes family members have to hold up X-ray films, making it difficult to see clearly. Therefore, while simple health consultations are convenient, for complex or critical cases, patients or their families may struggle to answer detailed questions or lack the necessary equipment. In such situations, assistance from community doctors is essential to facilitate effective remote consultations.

**12. So, the issues arise from both equipment limitations and technological constraints?**

Yes.

**13. In your opinion, how can digital-intelligent healthcare be improved in the future, particularly in clinical applications or patient procedures?**

From the patient's perspective, China's policy of promoting a tiered healthcare system calls for more comprehensive community services. For complex cases, remote consultations with larger hospitals could be conducted with the support of community doctors. From the doctor's perspective, safety assurances need to be strengthened. Information obtained through remote diagnosis may differ from reality, which involves medical safety concerns and requires systemic safeguards, including further refinement of referral processes. Additionally, while remote consultations are convenient, I believe they should not excessively encroach on doctors' non-working hours.

**14. Perhaps a common issue in Beijing and nationwide is the relatively weaker medical resources at the community and primary care levels. Could digital-intelligent healthcare be more closely integrated with the development of primary care?**

Yes. Patients can first visit community hospitals, which can also help doctors at major hospitals better understand the actual condition of the patients.

**15. You mentioned earlier the issue of doctors working overtime or extending their hours. What policy improvements do you think are needed in this regard?**

Although doctors’ primary motivation is to serve patients, sustainability requires consideration of time, institutional support, and economic incentives. For instance, in remote consultations, patients may record conversations without the doctor’s knowledge. If such recordings are maliciously edited and shared online, it could easily lead to public misunderstanding and increase risks for doctors. These factors must be taken into account. Without economic support, motivation may also be lacking. That said, the broader trend toward digital-intelligent healthcare is undoubtedly moving in the right direction—it’s just that many aspects still require refinement as we continue to explore and develop it.
