# Supplementary material for: Research on the implementation path of digital-intelligent healthcare based on the TAM model from the perspective of high-quality development
Source: BMC Health Serv Res. 2026 Mar 27;26:646. doi: 10.1186/s12913-026-14433-1 (PMC13151098; doi:10.1186/s12913-026-14433-1)
Supplement: Supplementary file 8 — Supplementary Material 8 [file 12913_2026_14433_MOESM8_ESM.docx]

Interviewee G: an elderly person

**1. What was your previous occupation?**

I was a farmer and started working in the fields at the age of 16.

**2. Do you currently live alone or with your children?**

I live with my spouse.

**3. What is the history of your cerebral infarction?**

I am currently in the recovery period. It has been a year and a half since the onset, and I did not undergo surgery. Recovery mainly relies on medication and self-directed exercises. I now exercise twice daily. For cerebral infarction, surgery is usually not necessary—recovery tends to be gradual. Some issues may persist for a long time, while others may improve quickly.

**4. Do you frequently go to the hospital?**

Not often. I was hospitalized for about a month before the May Day holiday and have not been to the hospital since discharge. My recovery primarily involves acupuncture, massage, and exercise.

**5. Are you currently using a smartphone?**

Yes, a Huawei phone.

**6. Do you usually shop using your phone? Do you use shopping apps like Taobao?**

Although the phone is convenient, I mainly shop offline at supermarkets and farmers' markets.

**7. Did you receive any high-tech medical treatments during your hospitalization?**

No, just regular medical care.

**8. Were you previously aware of any high-tech medical advancements?**

No, I only learned about it when you mentioned it. I have used large medical equipment a few times during hospital check-ups, such as CT scans.

**9. How did you make hospital appointments? Did you book them yourself?**

Appointments were made via phone. At that time, I couldn’t book one myself due to my condition, so my daughter-in-law made the appointment for me.

**10. Do you find this online appointment system convenient?**

Yes, it’s convenient. Booking via phone saves time from waiting in line.

**11. You mentioned earlier that your left leg is somewhat inconvenient. Suppose there was a high-tech wearable device that could allow you to walk freely—would you be willing to try it?**

I haven’t really seen such a device, but if the price is reasonable, I would be open to it.

**12. Was your spouse proficient in making the appointment for you? Did she look into the appointment process beforehand?**

She knows how to use these kinds of programs. We’re not particularly old yet, so using smartphones isn’t a problem for us. Right now, we mainly use the phone for making calls, sending WeChat messages, and shopping—in practice, it’s not much different from using a basic senior-friendly phone.

**13. If relatives or friends around you needed to make a medical appointment, would you recommend this online appointment method to them?**

I’m not sure. Sometimes when my daughter needs to buy common medicines, she buys them online and has them delivered home, which is quite convenient.

**14. You seem quite positive about online appointments and devices like CT scanners. If both online and offline appointment options were available to you, would you still choose online booking?**

I would still prefer online appointments. Sometimes when we go to the hospital in the morning, the queues at the window are very long. Nowadays, there are also self-service registration machines, so you can register directly—though I haven’t used one myself.

**15. Are you open to your children helping you buy medicine online?**

Yes, but I don’t know how to do it myself—my children do. Right after I got sick, it was always my daughter-in-law or children who handled these things. I generally just wait for treatment and don’t try to learn or use these tools on my own.

**16. Are you concerned about online privacy and security issues?**

No, there isn’t much “privacy” nowadays anyway. I still have a lot of trust in these online tools.

**17. What do you think about the service quality of current high-tech devices?**

The service quality is quite good. For example, when shopping at the supermarket, sometimes you don’t even need a cashier—you can use the self-checkout machine to pay by yourself.

**18. Would you like to see these online tools become more sophisticated and widespread?**

Yes, I hope so. Mainly for the convenience of people like us, or even older seniors. At least when we go to the hospital with our children, they can use these tools.

**19. If you had a way to learn more about such devices in the future, would you be willing to?**

Yes, I would. I would even proactively consider wearing smart devices to ensure my money is well spent. After getting sick, my main mindset is to recover as quickly as possible.

**20. Would you accept a robot replacing a surgeon to perform an operation?**

I would accept it if it’s safe, because for such devices to be successfully promoted in the future, their quality must be up to standard. Some relatives or friends who have used high-tech devices might recommend them to me, but I’m not particularly interested.
