# Supplementary material for: Research on the implementation path of digital-intelligent healthcare based on the TAM model from the perspective of high-quality development
Source: BMC Health Serv Res. 2026 Mar 27;26:646. doi: 10.1186/s12913-026-14433-1 (PMC13151098; doi:10.1186/s12913-026-14433-1)
Supplement: Supplementary file 9 — Supplementary Material 9 [file 12913_2026_14433_MOESM9_ESM.docx]

Interviewee H: an elderly person

**1. How often do you go to the hospital?**

About once every two months, mainly to get my regular medication.

**2. Do you undergo regular health check-ups?**

Rarely, only occasionally. Many check-ups don’t detect actual problems and aren’t very suitable for people like us. I mainly go to major hospitals to get blood tests done, like checking blood lipids and similar items.

**3. Have you used online appointment booking when visiting the hospital?**

Nowadays, I always book appointments via my phone.

**4. Have you encountered any smart devices when measuring your blood sugar?**

No, I haven’t seen any. Whether it’s fingertip blood sampling or venous blood drawing, I haven’t come across such devices. I get my blood tested about once a year.

**5. There are now remote health monitoring devices, which are closely related to “digital-intelligent healthcare”—primarily integrating artificial intelligence with medical care. Have you used any similar devices?**

No, I haven’t. I previously considered insulin injections. I was diagnosed with high blood sugar in 2003, but even the smallest dose of insulin was not tolerable for me, so I now rely mainly on medication, taken twice a day.

**6. Do you think such online methods make healthcare more convenient for you?**

Yes, it’s convenient. Sometimes I also search online to understand the reasons behind my condition. You just need to click, and the results come up—it’s very handy.

**7. How do you find the ease of use of such operations? Do you encounter any difficulties during the process?**

No, I can handle it all.

**8. Are you willing to recommend this method to people around you?**

Yes, I often share it with my spouse and teach him how to buy and order medicine online.

**9. Are you concerned about data security?**

No, I still have a high level of trust in it.

**10. Do your children support you in using these methods?**

Yes, my son and grandson are very supportive.

**11. What are your expectations for the future development of digital-intelligent healthcare?**

One inconvenience I’ve noticed is that sometimes asking the same question online can lead to different answers, and the responding doctors may change frequently. Even though the general guidance is correct, it can still be somewhat confusing.
